# Improving school management in low and middle income countries: A systematic review^☆^

**DOI:** 10.1016/j.econedurev.2023.102464

**Published:** 2023-12

**Authors:** Gautam Anand, Aishwarya Atluri, Lee Crawfurd, Todd Pugatch, Ketki Sheth

**Affiliations:** a Global School Leaders, United States of America; b J-PAL South Asia, India; c Center for Global Development, United States of America; d Oregon State University, United States of America; e University of Tennessee, United States of America

**Keywords:** School management, Principals, School leaders, Literature review

## Abstract

Improving school quality in low and middle income countries (LMICs) is a global priority. One way to improve quality may be to improve the management skills of school leaders. In this systematic review, we analyze the impact of interventions targeting school leaders’ management practices on student learning. We begin by describing the characteristics and responsibilities of school leaders using data from large, multi-country surveys. Second, we review the literature and conduct a meta-analysis of the causal effect of school management interventions on student learning, using 39 estimates from 20 evaluations. We estimate a statistically significant improvement in student learning of 0.033 standard deviations. We show that effect sizes are not related to program scale or intensity. We complement the meta-analysis by identifying common limitations to program effectiveness through a qualitative assessment of the studies included in our review. We find three main factors which mitigate program effectiveness: (1) low take-up; (2) lack of incentives or structure for implementation of recommendations; and (3) the lengthy causal chain linking management practices to student learning. Finally, to assess external validity of our review, we survey practitioners to compare characteristics between evaluated and commonly implemented programs. Our findings suggest that future work should focus on generating evidence on the marginal effect of common design elements in these interventions, including factors that promote school leader engagement and accountability.

## 1 Introduction

Good school management has consistently and robustly been associated with better student learning outcomes (Bloom et al., 2015, Crawfurd, 2017, Tavares, 2015). In addition, evidence from the United States and Canada show that teachers’ professional environment affects student achievement (Jackson, 2013, Jackson and Bruegmann, 2009, Papay et al., 2020, Papay et al., 2012) and principal value-added estimates are high (Branch et al., 2012, Dhuey and Smith, 2018, Grissom et al., 2015), underscoring the potential importance of the principal and school management. In low- and middle- income countries (LMICs), both school management quality and student learning outcomes are poor (Azevedo et al., 2022, Bloom et al., 2015, Lemos et al., 2021). Improving the productivity of key personnel in school systems, such as school leaders, may be a promising direction for raising student learning. As a result, there is growing attention from policymakers to interventions that target school leaders and their management of schools.^1^

This paper reviews and synthesizes the emerging evidence on LMIC school management and the efforts to improve it. Our primary research question is:

*How effective are school management interventions at improving student learning in low and middle income countries?*

In answering this question, we also address two auxiliary questions:

*Who are school leaders and what decisions do they make?*

*How do the characteristics of evaluated school management programs compare with programs being implemented by practitioners?*

Answers to these questions are critical to understanding whether the association between management and student learning can be leveraged in targeted interventions that increase school performance. Such efforts face considerable challenges, including that the causal chain required from management improvements to student learning is longer than interventions that target teachers or other inputs that directly interact with students (Ganimian and Freel, 2020, Mbiti, 2016). However, even if management interventions yield smaller improvements, they hold great promise, because the actions of school leaders can spur improvements throughout an entire school, resulting in a more cost-effective intervention (Fryer, 2017, Grissom et al., 2021). This cost effectiveness is especially important for low- and middle-income countries, where student learning is a global priority, and resources and institutional capacity are generally more constrained.

Designing an effective intervention targeting school leaders should take into consideration the characteristics of those principals and the scope of their authority.^2^ We therefore start with descriptive evidence on the characteristics and responsibilities of school leaders, using data from large-scale, multi-country surveys in middle and high income countries. We highlight that school leaders in middle-income countries generally self-report having less responsibility for key decisions in their school relative to their counterparts in high-income countries. In the majority of schools, in both high- and middle-income countries, school leaders self-report *not* actively being involved in salary decisions and course content. We find that educational attainment, specialized training, and experience as a teacher is generally higher in high-income countries, though overall experience is similar. When augmenting our data with additional surveys which include low-income countries, we also find that female representation among principals is significantly lower in low- and middle-income countries. We also review advancements in measuring school management (Bloom et al., 2015, Leaver et al., 2019).

We then systematically review evaluations of interventions to improve school management that estimate causal effects on student learning. We identify 20 experimental or quasi-experimental evaluations of school management interventions targeting principals, 15 of which are based in low and middle income countries.^3^

Most of these studies find statistically significant improvements on school management or related proxies, and most fail to similarly detect statistically significant positive effects on student learning. However, when we aggregate studies through a meta-analysis, we find an overall positive and statistically significant average effect of 0.033 standard deviations on learning outcomes. This effect is driven by greater weight placed on results which are more precisely estimated, which tended to be positive. The meta-analysis results are robust to excluding any individual study, confirming that results are not driven by a specific intervention or context. Although relatively small in magnitude, the application of this effect to an entire school could imply considerable cost-effectiveness, particularly when contrasted with alternative interventions such as teacher training which are limited to individual classrooms.

Using data on program features, we test for explanations of our main finding. We fail to detect a statistically significant relationship between the scale of a program and its effectiveness, suggesting the positive effects are not driven by smaller interventions that generally have more control over implementation. The lack of correlation between scale and effect size is potentially encouraging for policy, because it suggests that programs of varying sizes may face common challenges. Moreover, we find no correlation between program intensity, measured in days of management training, and effectiveness. This is another encouraging finding, given constraints on school principals’ time. These findings are correlational and tentative due to limited data, but suggest fruitful directions for future research.

Through a qualitative assessment of studies included in our review, we identify three common barriers to program effectiveness. First, many studies report low take-up of management interventions by principals. Second, lack of incentives or structure prevented school leaders from implementing the intended improvements. And third, management improvements must be relatively large to be effective, given the lengthy causal chain from management practices to student learning. Implementation gaps at any stage may result in an ineffective intervention.

To understand the external validity of this evidence, we collect primary data to compare the programmatic details of evaluated interventions with interventions implemented by practitioners. We adapt the “in-service teacher training survey instrument” developed by Popova et al. (2022) to survey evaluators and practitioners of school management programs.^4^ We find that the evaluated programs included in our systematic review are similar in intensity (i.e., program time) and trainer-to-beneficiary ratios as those implemented by practitioners, and are similar to NGO programs in terms of quality practices included in the intervention. However, we also find that the evaluated programs cover fewer schools and are more expensive, suggesting limited external validity along these dimensions.

We make three main contributions to the literature. First, we document characteristics and decision-making authorities of school principals using large, multi-country datasets. Our descriptive analysis of principals complements studies measuring school management practices worldwide (Bloom et al., 2015, Leaver et al., 2019).

Second, our systematic review on improving school management in low and middle income countries presents the most comprehensive and current review of which we are aware. Our review builds on earlier efforts to understand the evidence on management and school leaders in schools. Grissom et al. (2021) reviews six well-executed studies in the United States to estimate the value-added of principals on student test scores. In a review of the literature on school leadership in the global south, Global School Leaders (Leaders, 2020) report the robustness of the correlation between school leadership and student learning, and review five impact evaluations of training school leaders (a subset of the papers we include in this review).^5^ We expand on their work by employing a systematic approach to identifying relevant studies on the causal effect of management focused interventions, and by conducting a meta-analysis of the included studies. We join a growing number of meta-analyses of results from impact evaluations in economics and international education (Bandiera et al., 2021, Castaing and Gazeaud, 2022, Jackson and Mackevicius, 2021, Kremer et al., 2022, Meager, 2019, Vivalt, 2020). A meta-analysis allows us to rigorously aggregate effect sizes into a single average, boost the power of individual studies through pooling, and test important dimensions of heterogeneity. A limitation of the approach is that it groups distinct school management interventions to estimate an average effect. Though the meta-analysis allows us to assess the importance of one factor (e.g., scale) at a time, even similar interventions in this review differ in non-trivial ways that a meta-analysis fails to account for. We therefore complement the meta-analysis with a qualitative description of the literature, focusing on idiosyncratic features of individual programs and studies.

Third, we document remaining evidence gaps by comparing the characteristics of programs evaluated using rigorous research designs with programs implemented at scale. Understanding these gaps is important for policymakers concerned with designing programs for impact and for researchers seeking to contribute more relevant evidence. This exercise builds on similar work reviewing current practice in teacher training (Popova et al., 2022) and school management training in Latin America and the Caribbean (Adelman & Lemos, 2021).

The rest of this paper proceeds as follows. In Section 2, we present descriptive evidence on school leaders and their responsibilities, describe how school management is measured, and discuss the broader literature on the relationship between management and productivity. In Section 3, we systematically review and meta-analyze the literature on school leadership interventions. In Section 4, we discuss data from our original survey of the characteristics of effective and at-scale programs. Finally, in Section 5, we conclude and provide direction for future research.

**Table 1:** Principal characteristics.

	LIC	MIC	HIC	diff(MIC-HIC)
Share of female school leaders (%)^a^	26.33	43.21	52.83	−9.62
Years as principal - Total		9.21	10.03	−0.82
Years as principal - in this school		6.76	6.80	−0.04
**Highest qualification (%)**^b^				
No degree or equivalent		6.51	3.91	2.59
Bachelor’s degree or equivalent		48.57	39.58	8.99
Master’s degree or equivalent		39.45	52.83	−13.37
**Educational leadership qualifications (%)**^b^				
Certificate or license in Ed leadership		70.34	68.27	2.07
Master’s degree or equivalent in Ed leadership		28.16	36.89	−8.73
**Policy on requirements to become a principal (%)**^c^				
Teaching experience		63.16	73.17	−10.01
Specialized leadership training		32.00	52.63	−20.63
Requirements changed in last 10 years (Primary school)		36.00	20.69	15.31
Requirements changed in last 10 years (Secondary school)		25.93	23.64	2.29
N (Countries)	11	18	38	

Notes:

a Data on school leader gender is taken from IPUMS, PASEC, SACMEQ, TALIS, and Service Delivery Indicator (SDI) surveys. This leads to data for 11 low-income countries, 37 middle-income countries, and 32 high-income countries. All other data is from the TIMSS 2019 School and Curriculum Questionnaires. This includes data from 18 Middle Income Countries (MIC): Albania, Armenia, Azerbaijan, Bosnia and Herzegovina, Bulgaria, Georgia, The Islamic Republic of Iran, Kazakhstan, Kosovo, Montenegro, Morocco, North Macedonia, Pakistan, Philippines, The Russian Federation, Serbia, South Africa, Turkey; and 38 High Income Countries (HIC): Australia, Austria, Bahrain, Belgium, Canada, Chile, Croatia, Cyprus, Czech Republic, Denmark, Finland, France, Germany, Hong Kong SAR, China, Hungary, Ireland, Italy, Japan, The Republic of Korea, Kuwait, Latvia, Lithuania, Malta, Netherlands, New Zealand, Norway, Oman, Poland, Portugal, Qatar, Saudi Arabia, Singapore, Slovak Republic, Spain, Sweden, United Arab Emirates, United Kingdom, The United States of America. The primary and secondary schools mentioned in the table are specific to the Grade 4 and Grade 8 data in the TIMSS dataset.

b Generated using country-level averages reported in the TIMSS dataset

c Generated using individual yes/no aggregate response for each country in the TIMSS dataset. The percentage reported here is specific to the TIMSS country sample and the country’s income-level classification according to the World Bank. Therefore, the sum of percentages will not add up to 100.

## 2 Who are school leaders and what do they do?

### 2.1 Characteristics of school leaders

Comparable data on school leaders across both low-, middle-, and high-income countries is limited, with data from low-income countries particularly scarce. Nonetheless, because of the importance of the exercise, in this section we collate data from a number of different sources, primarily focusing on differences between middle- and high-income countries. Overall, we find higher levels of education, experience as a teacher, and specialized leadership training among high-income principals relative to middle-income principals, though overall education and experience is high across the board. We similarly find that higher-income country principals self-report greater levels of responsibility.

We first focus on the 2019 Trends in Math and Science Survey (TIMSS), which covers 18 middle-income and 38 high-income countries (summarized in Table 1).^6^ Principals in high- and middle-income countries have similar levels of experience (nine to ten years), almost seven of which are at their current institution. While most school leaders are highly educated, those in high-income countries are more likely to hold a masters degree. Only a minority do not have any tertiary degree. Nearly 70 percent also have a specific certificate or licence for educational leadership, in both middle-income and high-income countries. High current levels of formal education may suggest that qualification requirements are not a likely pathway for improving management practices.

These measures of experience and education may hide subtler differences in human capital. For example, though overall experience is similar, experience as a teacher and specialized leadership training as part of the path to becoming a principal is more common in high income countries. In the majority of countries teaching experience is a requirement to become a school leader, but specialized leadership training is not. The survey also highlights that requirements for being a principal change in many countries over time, both for primary and secondary schooling. Middle income countries are more likely to have policy changes on the requirements of being a principal in the last ten years.

Combining data from five different sources,^7^ we find that school leaders in low-income countries (LICs) are much less likely to be women, with women comprising only 26 percent of leaders in LICs, compared to 53 percent in high-income countries. Given evidence of role model effects and gendered networks (Beaman et al., 2012, Golan and You, 2021, Kipchumba et al., 2021, Muralidharan and Sheth, 2016, Nguyen, 2008, Serra, 2022), increasing representation of women in school leadership may increase school performance, especially among female students and teachers.

### 2.2 The decisions school leaders make

School leaders administer schools, with typical responsibilities including financial decisions, academic oversight, and management. In this section, we present comparative cross-country data from the 2015 Programme for International School Assessment (PISA) survey on school leader autonomy over key decisions (Table 2). As with any self-reported data, responses may reflect systematic cultural differences in understanding and norms on answering questions across countries, and what principals may report may not reflect reality. Though self-reported data is the best alternative in the absence of objective and independent measures on a similar scale, we note caution in interpretation.

Across several key tasks, school leaders in high-income countries report having more autonomy than leaders in middle-income countries (Table 2, Columns 1–2; similar to patterns found by Hanushek et al., 2013). For example, regarding teacher management, principals in high-income countries are 23 percentage points more likely to have responsibility for selecting teachers to hire, and 15 percentage points more likely to have responsibility for firing teachers, compared to middle-income countries. Turning to academic policies, principals in middle-income countries are 12 percentage points less likely to be responsible for setting the school budget, and 23 percentage points less likely to be responsible for budget allocations within the school. Only a minority of principals in middle-income countries are responsible for curricular decisions such as choosing textbooks, course content, or which courses to offer. For each task listed in Table 2, principals in middle-income countries are less likely to self-report that they have considerable responsibility relative to their high-income country counterpart.

Furthermore, in middle-income countries, the majority of principals generally report that they do not have considerable responsibility over a larger range of critical decisions in teacher policy nor in academic policy. Relative lack of authority by principals to make decisions in middle-income countries may be an important intermediary in the relationship between a school leader’s management skill and school performance.

Table 2 also highlights general patterns of school leader responsibilities across both middle and high income countries. In both high and middle-income countries, salaries and course content and material are generally out of the purview of school leaders. For example, only around one in five school leaders has responsibility for setting salaries, excluding them from a key personnel decision. This suggests that targeting these areas in interventions with school leaders may not be relevant.

In contrast, in both middle- and high-income countries, at least 40 percent of school leaders decide budget allocations, set the school budget, select which teachers to hire and fire, set disciplinary policy, and approve admissions. Given the higher level of authority along these dimensions, they may be particularly promising areas of focus for school leadership interventions.

Table 2, Columns 3–7 document differences by region. We see relatively similar ranges across the different regions, but countries in Latin America typically have less autonomy than countries in East Asia, Europe and Central Asia, and the Middle East and North Africa.

**Table 2:** School leader autonomy over key decisions.

		(1)	(2)	(3)	(4)	(5)	(6)	(7)
		MIC	HIC	EAP	ECA	LAC	MENA	NA
1	Selecting teachers to hire	45	68	60	69	32	48	81
2	Firing teachers	41	56	46	61	26	45	44
3	Setting starting salary	19	21	20	24	14	20	4
4	Setting salary rises	21	23	27	26	13	22	4
5	Setting school budget	45	57	62	56	37	49	45
6	Deciding budget allocations	53	76	74	71	46	64	87
7	Setting disciplinary policy	50	71	70	64	55	59	84
8	Setting assessment policy	35	60	62	51	44	43	66
9	Approving admissions	62	72	72	68	61	68	77
10	Choosing textbooks	19	31	30	26	22	27	42
11	Choosing course content	18	26	29	23	20	23	25
12	Choosing which courses	30	63	61	54	36	36	87
	N(Countries)	24	44	11	37	10	8	2

Note: This table shows data on the share of principals who self-report that they have “considerable responsibility” for the listed tasks (Question 10 from the 2015 PISA School Questionnaire). MIC indicates Middle-Income Countries, HIC High-Income Countries, EAP East Asia and Pacific, ECA Europe and Central Asia, LAC Latin America and Caribbean, MENA Middle East and North Africa, and NA North America.

### 2.3 School leader management skills

What does school management mean? This subsection describes how management is measured, focusing on recent advances. We then briefly review evidence on the relationship between measured management practices and organizational performance, particularly in schools and other public sector institutions, and in low- and middle-income countries.

#### 2.3.1 Measuring management

Economic theory considers management a technology for allocating resources within an organization. In the canonical production function relating capital (K) and labor (L) inputs to output (Y), Y=A∗F(K,L), management can be considered a component of A, shifting the productivity of existing inputs.

Prior research has focused on specific aspects of school operations, such as teacher absenteeism (Chaudhury et al., 2006) or teacher time-on-task (Stallings, 1977, Stallings and Mohlman, 1988), which have been perceived by some as indicative of management (e.g., a high prevalence of absent teachers may suggest poor management). Increasingly, these measures are complemented by direct measures of school management. A key innovation was the introduction of the World Management Survey (WMS), an inventory of management practices gathered through comprehensive, structured interviews with managers (Bloom et al., 2014, Bloom and Van Reenen, 2007, Bloom and Van Reenen, 2010, Scur et al., 2021). The WMS “measure[s] management practices in three broad areas: (1) monitoring — how well do companies monitor what goes on inside their firms and use this for continuous improvement?; (2) targets — do companies set the right targets, track the right outcomes, and take appropriate action if the two are inconsistent? (3) incentives — are companies promoting and rewarding employees based on performance, and trying to hire and keep their best employees?” (Bloom & Van Reenen, 2010 p. 207).

Originally designed for private sector firms, the WMS was later adapted for public sector organizations such as schools (Bloom et al., 2015), and for developing countries (Lemos & Scur, 2016). This Development WMS (D-WMS) now includes 23 management domains, split into operations management (14 domains) and people management (9 domains). Within each domain, the D-WMS maps qualitative information on management practices gathered from interviews into a numerical score from 1 to 5, in half-point increments. Tables A3–A4 list all D-WMS domains.

#### 2.3.2 The importance of management skills across sectors

The WMS, D-WMS, and other measures of managerial skill predict organizational performance across many settings. In the private sector, better management correlates with firm performance in developed (e.g., Bloom & Van Reenen, 2007) and developing countries (e.g., Adhvaryu et al., 2019). Experimental interventions to improve firm management in LMICs have succeeded in several contexts, including textile firms in India (Bloom et al., 2013), small- and medium-sized firms in Mexico (Bruhn et al., 2018), and auto parts manufacturers in Colombia (Iacovone et al., 2022). In other settings such as tailors in Ghana, sustaining short-term improvements has proven difficult over longer horizons (Karlan et al., 2015).

Management skills have also been found to be important in the public sector, including in LMICs, where incentive structures and institutional constraints differ (Finan et al., 2017). For instance, management practices correlate with task and project completion rates in the Nigerian civil service (Rasul and Rogger, 2018, Rasul et al., 2021), and with medical treatment and infection control adherence in Tanzania (Powell-Jackson et al., 2022). Management practices for public service delivery are also malleable; an RCT providing improvement plans and implementation support in the Nigerian health sector improved their practices (Dunsch et al., 2017).

#### 2.3.3 Management in education

Descriptive evidence consistently demonstrates the importance of management in education. In a systematic review of the US literature, Grissom et al. (2021) found six well-executed studies which estimated principal value-added (VA) on student test scores (Bartanen, 2020, Branch et al., 2012, Chiang et al., 2016, Dhuey and Smith, 2018, Grissom et al., 2015, Laing et al., 2016). These studies estimate principal VA, measured as mean deviations of student test scores at a principal’s school from scores predicted from observable characteristics of the student and school. Averaging across these studies, a one standard deviation (sd) increase in principal VA corresponds to mean student test score gains of 0.13 sd in math and 0.09 sd in reading. These gains fall only slightly below the benchmark estimates of teacher VA of 0.16 sd in math and 0.12 sd in English (Chetty et al., 2014). If treated as causal effect estimates, replacing a principal at the 25th percentile of the VA distribution with one at the 75th percentile would increase average learning by about one-third of a school year. Moreover, teacher VA applies only to the teacher’s classroom, but principal VA applies to all students in a school. The scope of a principal’s influence led (Grissom et al., 2021) to conclude, “[I]f a school district could invest in improving the performance of just one adult in a school building, investing in the principal is likely the most efficient way to affect student achievement” (p. 40).

However, principal value added does not necessarily equate to management practices nor reflect causal effects of the principal. Indeed, recent work has called into question the validity of principal VA estimates, which do not rely on direct measures of management, as measures of principal effectiveness. Using data from three US states, Bartanen et al. (2022) find low correlation in estimates of a principal’s VA over time. They interpret this finding as evidence that principal VA consists largely of transient factors outside of a principal’s control. Even when principal VA is taken at face value as a measure of principal effectiveness, effective principals might rely on factors such as personal charisma rather than management skill.

Beyond principal VA, stronger management practices generally correlate with higher student performance. For instance, in a descriptive study of US charter schools, Dobbie and Fryer (2013) find that management practices such as frequent teacher feedback and using data to guide instruction were associated with higher student performance, whereas input measures such as class size and expenditure per student were not. Other studies from the US suggest the importance for student achievement of the professional environment for teachers (Jackson, 2013, Jackson and Bruegmann, 2009, Papay et al., 2020, Papay et al., 2012), an area likely influenced by school principals. A systematic review of studies from high-income countries finds correlations between principal management behaviors and student outcomes, as well as teacher well-being and practice (Liebowitz & Porter, 2019).

This relationship between management practice in schools and student performance extends internationally. The WMS and D-WMS have also been administered and shown to correlate with student performance in India (Lemos et al., 2021), Nigeria (Lipcan et al., 2018), and Uganda (Crawfurd, 2017). Bloom et al. (2015) administered the WMS to more than 1800 schools in eight countries. They find a one standard deviation increase in management practices was associated with 0.2–0.4 sd increases in test scores. Decomposing the variation in school management revealed about half was due to differences across countries and half within. Overall, management practice scores are much higher in high-income countries. Within countries, they found autonomous government schools, such as US charters and UK academies, tend to have higher management scores than standard public schools. Leaver et al. (2019) construct an index of management practices based on the WMS framework, using self-reported data across 65 countries from the Programme for International Student Assessment (PISA). As in other studies, they find strong positive correlations between management practice and student performance. The association between management practices and school performance is therefore widespread and robust.

## 3 Improving school management in developing countries

### 3.1 Systematic review protocol

In the previous section, we outlined the evidence on the importance of school management for student outcomes. However, this leaves open the question of the effectiveness of interventions that target school leaders to improve school management.

In this section, we systematically review the evidence on efforts to improve school management. We screened studies based on three dimensions: (1) program content, (2) study methodology, and (3) student learning outcomes. For program content, we consider interventions which engage school principals directly and targeted improving the management of the school, for instance through management training or the development of school improvement plans.^8^ For study methodology, we include only randomized control trials or quasi-experimental research designs to estimate causal effects on learning outcomes. The quasi-experimental research designs considered include regression discontinuity or regression kink designs; difference in differences; event studies; instrumental variables estimation; and synthetic control. For student learning outcomes, we focus on student test scores, scaled to the standard normal distribution. This approach follows the norm in the economics of education, notwithstanding the limitations to the comparability of different assessments (Bertling et al., 2023).

We searched Google Scholar on 18th August 2022 for articles containing the terms (“school leader” OR “school principal” OR “headteacher” OR “school management”) AND (“training”) AND (“student achievement” OR “learning outcome” OR “test score”) AND (“impact evaluation” OR “field experiment”). This search resulted in 2558 unique results.^9^ We also considered eight additional studies we learned of through other sources, such as social media or colleagues.

We manually screened the titles of these studies and reduced the number to 80 potentially relevant studies. Multiple co-authors then independently reviewed the full text of these papers and removed papers that did not meet the above criteria for content, methodology, and student learning outcomes. If a paper was marked differently, then the paper was discussed until we reached consensus.^10^ We additionally included 8 studies known to the authors that met the criteria, but did not come up in the original search. We also reviewed World Bank Reports on projects involving school leaders and the list of programs in Muralidharan and Singh (2020) to ensure additional evaluations meeting the criteria were not missed.

This resulted in 20 unique studies that fit our criteria (see Figure A1 for a summary flow diagram for this process.). Table 3 lists the country, type of school assessed, level of school assessed, the country’s income level, and the methodology of evaluation for each study used in our meta-analysis. The majority of the comparisons are identified using a randomized controlled trial (RCT; 16 of 20 studies). We did not restrict our search process by country, and so our final sample includes five studies from the United States. However, the main results presented are robust to omitting these studies. Within each study we extract the authors’ preferred estimate for impact on student learning per academic subject, from as many time points as are presented (several studies estimated effects after one year and after two years, in which case we include both sets of estimates). Overall, we have 56 estimates, of which 27 are in mathematics, and 29 in language. Twenty-four estimates are measured after one year, five after 1.5 years, 17 after two years, six after three years, two after 3.5 years, and two after four years (see Table A2).

**Table 3:** Overview of studies.

Study	Country	School level	Schools trained	Implementer	Method
Aturupane et al. (2022)	Sri Lanka	Sec	36	Government	RCT
Beg et al. (2021)	Ghana	Pri	210	Government	RCT
Blimpo et al. (2015)	Gambia	Pri	90	Government	RCT
de Barros et al. (2019)	Brazil	Sec	1732	NGO	RCT
de Hoyos et al. (2020)	Argentina	Pri	100	NGO & Gov	RCT
de Hoyos et al. (2021)	Argentina	Pri	105	NGO & Gov	RCT
Devries et al. (2015)	Uganda	Pri	21	NGO	RCT
Fryer (2017)	US	Pri/Sec	58	Government	RCT
Ganimian and Freel (2020)	Argentina	Pri	100	NGO	RCT
Garcia-Moreno et al. (2019)	Mexico	Pri	98	Government	RCT/DD
Garet et al. (2017)	US	Pri/Sec	63	Government	DD
Jacob et al. (2015)	US	Pri	62	Government	RCT
Kraft and Christian (2022)	US	Pri/Sec	123	Government	RCT
Lassibille et al. (2010)	Madagascar	Pri	303	Government	RCT
Lohmann et al. (2020)	Guatemala	Sec	2057	Government	RCT
Muralidharan and Singh (2020)	India	Pri	1774	Government	RCT
Romero et al. (2022)	Mexico	Pri	1198	NGO & Gov	RCT
Smarrelli (2021)	Peru	Pri	2650	Government	RD
Steinberg and Yang (2022)	US	Pri/Sec	642	NGO	DD
Tavares (2015)	Brazil	Pri/Sec	221	Government	RD

Note: All studies focus on public schools with the exception of Lohmann et al. (2020) which includes both Public and Private schools. School Level: Pri indicates primary; Sec indicates secondary. Method: RCT indicates Randomized Controlled Trial, DD indicates Difference-in-Difference, and RD Regression Discontinuity.

### 3.2 What are school leader/school management interventions?

Interventions that target improving the management of a school through the principal can cover a significant range of different elements and contexts. In Table 4, we provide details on the interventions used in this review.

**Table 4:** Study details.

Study	Training focus	P	F	I	C	M	O	Who else was trained
Aturupane et al. (2022)	How to prepare school improvement plan. Also increased school decision-making, allowed schools to raise funds locally.	Y	N	N	N	N	Y	Teachers and community representatives

Beg et al. (2021)	People management practice; differentiated instruction.	N	N	N	N	Y	Y	.

Blimpo et al. (2015)	How to prepare school improvement plan. Focus on six areas; (1) community participation, (2) learner welfare and school environment, (3) curriculum management, (4) teaching and learning resources, (5) teachers’ professional development, (6) leadership and management.	Y	Y	Y	N	Y	Y	Teachers and community representatives

de Barros et al. (2019)	Goal alignment and data use in planning.	Y	Y	N	N	N	N	Regional leaders and supervisors

de Hoyos et al. (2020)	How to prepare school improvement plan, conduct classroom observations and give teachers feedback, and understand effective teaching practices in math and language. Included online dashboard to monitor school improvement plan.	Y	N	Y	Y	N	N	.

de Hoyos et al. (2021)	Understanding standardized assessment results, school improvement plans, and quality assurance mechanisms.	N	N	Y	Y	N	N	Teachers

Devries et al. (2015)	School violence: setting goals, developing action plans with specific dates for deliverables, encouraging empathy by facilitating reflection on experiences of violence, providing new knowledge on alternative non-violent discipline, and providing opportunities to practise new behavioral skills. Included follow-up visits and support by NGO staff.	Y	N	N	Y	Y	Y	Two staff and two student “protagonists”

Fryer (2017)	Lesson planning, data-driven instruction, and teacher observation and coaching.	N	N	Y	Y	N	N	.

Ganimian and Freel (2020)	Six week course on (1) organizational development, (2) technological integration, (3) innovation in the curriculum, (4) improving teaching and learning, (5) develop relationships with the community, (6) teacher professional development.	N	N	N	Y	N	N	.

Garcia-Moreno et al. (2019)	How to prepare school improvement plan.	Y	Y	N	Y	Y	Y	Teachers and community representatives

Garet et al. (2017)	Performance feedback for principals and teachers.	N	N	N	Y	Y	Y	Teachers

Jacob et al. (2015)	Instructional leadership, with 21 principal responsibilities empirically linked to student test scores, including focus on curriculum, instruction, and data.	N	N	N	Y	N	N	.

Kraft and Christian (2022)	How to provide effective feedback to teachers.	N	N	N	N	N	Y	Vice-principals

Lassibille et al. (2010)	Making a School Improvement Plan. Use of workflow templates including teaching guidebooks, management tools, school report card produced with admin data.	Y	N	Y	N	Y	Y	District managers, teachers, community representatives

Lohmann et al. (2020)	Light-touch - one session providing ’rules of thumb’ guidance (informed by Fryer (2017)) on (1) lesson observation, (2) data-driven instruction, (3) teacher observation and coaching.	N	N	N	N	Y	N	.

Muralidharan and Singh (2020)	How to prepare school improvement plan and conduct school assessment. Quarterly follow-up by Cluster Resource Coordinator.	Y	N	Y	Y	N	Y	Cluster Resource Coordinators (supervise ∼40 schools each)

Romero et al. (2022)	Collect and use data to monitor students’ basic numeracy and literacy skills and provide teachers with feedback on their teaching style.	N	Y	N	N	N	N	.

Smarrelli (2021)	School violence: identification, reporting, management of incidents, and violence reduction strategies. Included offsite workshops, follow-up visits, and group learning sessions.	N	N	N	Y	N	Y	Teacher representative

Steinberg and Yang (2022)	Strategic planning, use of data to identify school needs.	N	N	N	N	N	N	.

Tavares (2015)	How to use student assessment data in goal-setting, planning, and monitoring.	Y	N	Y	Y	N	Y	Senior teachers

Note: **P**: School Improvement Plan; **F**: Funding; **I**: Information on school Performance, **C**: Customized Feedback; **M**: Materials; **O**: Includes Other Trainees.

Responses: **Y**: Yes; **N**: No;

#### 3.2.1 Definitions

In Table 4 we indicate whether the intervention provided materials, information on school performance, included a school improvement plan, provided training to the principal, included monetary funds, incorporated customized feedback, or included other key personnel. We also note other key details.

*Training Focus* provides a brief overview of the training or workshop provided to school leaders. Some interventions included training teachers or other key personnel in the school, but we only describe trainings if the intervention trained the school leader. Professional development was a common term used in describing the training provided and could cover a wide range of items. Note that the focus on school management implied that in the majority of cases, the professional development incorporated elements of improving managerial skills. If the intervention described a workshop, we interpreted this as training. Trainings varied widely in scope and intensity.

*School Improvement Plan* (P) indicates that the intervention focused on developing and implementing a school improvement plan.

*Funding* (F) indicates whether the intervention included any grant for the school.

*Information on School Performance* (I) indicates that the intervention provided school leaders with information on their school, such as student learning assessments. In one case, the intervention tasked the school leaders to collect this information. But in all other cases, this information was generally provided by an external entity.

*Customized Feedback* (C) is indicated if the intervention included providing sessions or feedback tailored to the specific school. For example, this may have included coaching or technical assistance in reviewing school assessments provided. This differs from the previous category in that the intervention actively uses school level information to engage the school leader, rather than passively providing the information.

*Materials* (M) indicates a program that explicitly noted that items such as templates, checklists, and so on, were provided. Some training programs may have included similar or extensive materials, but were not included in the intervention description and so are not noted here. We generally observe a study discussing materials if that was a key component of the intervention, which is often the case when the training component is relatively low intensity.

*Includes Others Trainees* (O) indicates if the intervention included incorporating key personnel other than the school leader, either at the more centralized level (e.g., district leaders), personnel within the school (e.g., teachers), or the broader community (e.g., parents). We indicate in *Who else was trained* whether other trainees were other teachers, parents, community members, or supervisors.

Table A2 provides details on the comparisons used in the meta-analysis. In the majority of cases, the comparison was relative to “business as usual”. In a minority of cases, the comparison group was given some component of the intervention being studied. In these cases, we confirm that the marginal effect measured still fits our program content criteria; i.e., the marginal program elements engage school principals directly and target improving the management of the school above and beyond the comparison group. In two studies, multiple comparisons were evaluated by the authors (Beg et al. (2021) and de Hoyos et al. (2021)). For these two papers, we use the business as usual comparison for discussion and the meta-analysis.

#### 3.2.2 Details of interventions

Table 3 highlights that studies focus largely on public schools (19 of 20 studies) and primary schools (17 of 20 studies). Eight studies include secondary schools.

In Table 4, we document that the most common method in which interventions aim to increase school management is through training principals on skills related to school management. The training focus and intensity vary across interventions, as well as the level of detail provided in each paper on the training curriculum. For example, in Guatemala, Lohmann et al. (2020) evaluate an extremely light touch program in which they offer a single training session and provide schools with a poster and a checklist from the training.

The next most common intervention design is to provide information on school performance. This includes information such as a school report card or diagnostic feedback on student learning. A school improvement plan is also a frequent method to improve school management. We also find that a relatively large number of interventions incorporate other key personnel in their intervention. Only three of the interventions included a training or feedback component tailored to the school, and four interventions included monetary support for management or other school improvements. Understanding the marginal effect of these common design elements may be a promising area for future research to identify what drives the effectiveness of school management interventions.

### 3.3 The effect of management programs on school management

The majority of interventions which measure behaviors of principals or teachers identified statistically significant effects, suggesting that programs were effective at changing practices. Due to the differences in management-related outcomes reported across studies, we do not run a meta-regression for principal behavioral outcomes and instead limit our meta-analysis to outcomes on student learning.

One study reports a 0.13 SD effect on the D-WMS index of management practices (Romero et al., 2022). Another finds a 0.3 SD improvement in a separate index of management practices inspired by the D-WMS (Beg et al., 2021). In addition, Tavares (2015) find increased engagement by principals in specific practices that occur in the D-WMS (including target-setting, monitoring student performance, using student performance data to adapt curriculum and plans), and Lassibille et al. (2010) see a 22 percentage point increase in the share of “well-managed schools” (defined as implementing seven tasks deemed to be “essential” by the Government: (1) keeping a register of enrollments, (2) signing off on a daily roll call, (3) regularly analyzing student absences, (4) reviewing student test results, (5) reviewing teacher absence, (6) reporting teacher absences to local government administrators, and (7) following-up with teachers on lesson planning).

Other papers report improvements along other dimensions in teacher or principal behaviors, though these effects were sometimes small in magnitude. For example, Blimpo et al. (2015) find positive effects on teaching practice, and student and teacher attendance; Lohmann et al. (2020) find an increase in the frequency of principals providing support to teachers; and Jacob et al. (2015) find reductions in teacher and principal turnover.

Though the majority of studies succeeded at changing practices, a handful of studies did not (e.g, Aturupane et al., 2022, Muralidharan and Singh, 2020). We discuss possible barriers to effectiveness in Section 3.5.

### 3.4 The effect of management programs on student performance: a meta analysis

#### 3.4.1 Random effects model

Table A2 documents each studies’ treatment effects on student learning. We conduct a meta-analysis that aggregates these studies’ findings into a mean effect on student learning, using a random effects model (Borenstein et al., 2021).^11^ The advantage of the random effects model is that it allows the true treatment effect to differ from study to study. The model assumes that there is a distribution of true effects with mean θ, and that the studies are a random sample from this distribution. The goal of the analysis is to provide an unbiased estimate of this mean treatment effect θ.

The analysis provides a summary effect (i.e., $\overset{ˆ}{\theta }$) that is a weighted average of the observed effect sizes on student learning (in standard deviation), with more precise estimates given more weight.^12^ ${\overset{ˆ}{\theta }}_{ij}$ is the observed effect for estimate i in study j, which is assigned a weight $W_{ij}$ based on the inverse of its variance, so more precise estimates have greater weight. Individual estimates differ from the overall mean θ due to the unobserved factors that drive the distribution of true effects (e.g. sample differences, etc.), and measurement error. The former has a constant variance, $\tau^{2}$, across all studies, while the latter’s variance, $v_{i}$, is specific to each study.

(1) $$\overset{ˆ}{\theta }=\overset{N}{\underset{i,j=1}{\sum }}{\overset{ˆ}{\theta }}_{ij}*W_{ij}$$

In our figures, we report the weight percent for a given estimate, which is calculated by

(2) $$W_{i}=\frac{1}{v_{i}+\tau^{2}}$$

We first show results by subject. Where we have multiple estimates for a subject within a study, we take the mean of the estimated effect and of the standard error (this is a conservative approach, assuming correlation of 1 between estimates and therefore providing no gain in precision and weight from having multiple underlying estimates). Weights are shown as a percentage of the overall average estimate.

This basic approach assumes that all estimates are independent. In order to properly account for the correlation between estimates within a study, we estimate a meta-regression with an estimator that is robust to unknown correlation between multiple estimates from the same study (Hedges et al., 2010). This estimator again uses inverse variance weights, in which $v_{•j}$ is the mean of the within-study sampling variances for each study j, $\tau^{2}$ is the estimate of the between-studies variance component (in a random effects model), but adds an additional term consisting of the number of effect sizes $k_{j}$ within each study j, and a constant ρ measuring the assumed correlation between all pairs of observed effect sizes within each study (which we again conservatively assume to be equal to 1; see Tanner-Smith and Tipton (2014) for more detail).

(3) $$W_{ij}=\frac{1}{\left\{\left(v_{•j}+\tau^{2}\right)+\left[1+\left(k_{j}-1\right)\rho \right]\right\}}$$

Turning to understanding heterogeneity between programmes, we estimate a meta-regression model by weighted least squares, in which observed effect sizes ${\overset{ˆ}{\theta }}_{ij}$ are related to m study covariates, 1 through M.

(4) $${\overset{ˆ}{\theta }}_{ij}=\beta_{0}+\beta_{1}X_{1ij}+\beta_{2}X_{2ij}+\cdots +\beta_{m}X_{Mij}+\epsilon_{ij}$$

#### 3.4.2 Results from the meta analysis on student learning

**Fig. 1:**
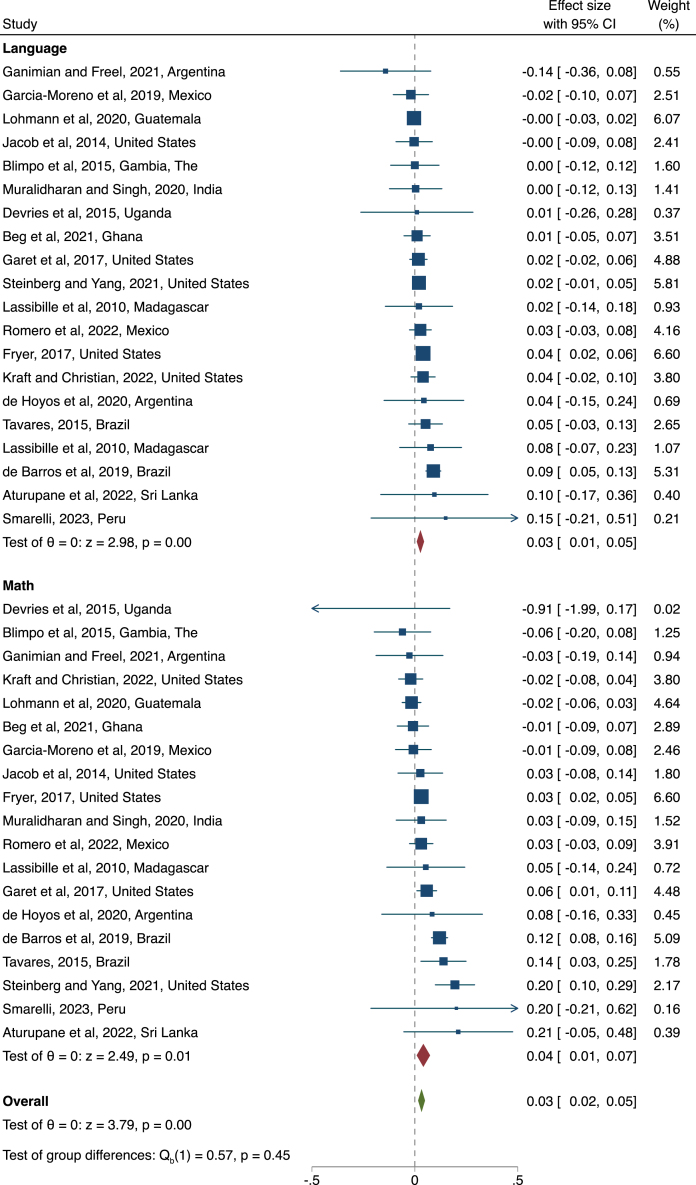
Summary of effect sizes, by subject Note: Squares indicate study effect sizes and solid lines indicate 95 percent confidence intervals. Square size is proportional to study weight, which is estimated based on the precision of the estimate. Red diamonds indicate sub-group mean effects, and the green diamond indicates the overall mean effect. Effect sizes and standard errors for each study are both calculated as the mean of individual estimates across different time periods within each study. This approach is conservative in assuming perfect correlation between estimates within each study, and so providing no increase in precision or weight for studies with multiple estimates (Borenstein et al., 2021). We show all individual estimates in Figure A2. (For interpretation of the references to color in this figure legend, the reader is referred to the web version of this article.)

The overall average effect from our meta-analysis is that these interventions targeting school leader’s management skills caused a statistically significant increase of 0.033 standard deviations (sd) in student test scores (Table 5). This estimate is based on 56 comparisons from 20 different studies. This result is similar in reading and mathematics (Fig. 1). Excluding studies from high income countries, we find a statistically significant effect of 0.03 sd on student learning. Expressed in learning-adjusted years of schooling (LAYS), which assumes annual gains of 0.8 sd in high-quality learning environments, this effect is equivalent to 0.033/0.8 = 0.04, or 4 percent of a quality school year (Angrist et al., 2020).

Another way to put this 0.033 sd magnitude in perspective is to consider how our estimate relates to observational estimates of the relationship between management practice and test scores. Bloom et al. (2015) finds a one standard deviation increase in D-WMS scores correlates with 0.4 sd higher test scores. Assuming a linear and unbiased relationship, an increase of 0.1 sd in the quality of management practices would yield learning gains of 0.04 sd.^13^ The magnitude therefore appears to match the positive, but moderate, increases in management quality in the studies we review.

A principal’s improved practices should also translate to benefits for the entire school. Improving test scores by 0.03 sd in a school of 600 yields an equivalent benefit, in total sd units gained, as improving scores by 0.2 sd for a teacher responsible for 100 of those students. The threshold determining an effective principal training program may therefore differ by an order of magnitude from an effective teacher training program.^14^ We can also compare our estimated effect size to the distribution of effect sizes from systematic reviews of all interventions in low- and middle-income countries. Across 234 studies, Evans and Yuan (2022) find a median effect size of 0.1 sd on learning. However amongst studies with the largest sample sizes, with which school training programmes are most comparable, the median effect size is 0.06 sd.

One possible concern is that studies that estimated positive effects may be over-represented in our sample due to publication bias. We find limited evidence of such a concern, based on inspection of a funnel plot (Figure A7) and the (Egger et al., 1997) asymmetry test. The funnel plot shows that estimates are symmetrical and mostly statistically significant, indicating a lack of publication or reporting bias. The linear (Egger et al., 1997) and nonlinear (Stanley & Doucouliagos, 2014) intercepts both adjust for any asymmetry, and produce estimates very similar to our main unadjusted estimate (Table A1).

Our results highlight the value of aggregating studies to understand the evidence on the effectiveness of interventions targeting school management on student learning. When considering each study individually, the majority of programs appear to not be effective; 43 of 56 estimates are not statistically significant at the 95 percent level. However this is primarily due to the majority of studies being under-powered. Just two studies are powered to achieve a minimum detectable effect of 0.033 standard deviations (we calculate the minimum detectable effect of each study ex-post as 2.8 × the standard error of the estimate). Yet, when aggregated, our meta-analysis suggests that on average there are positive gains in student test scores. The analysis is not driven by a single study, as the results remain robust to leaving out any one individual study (see Figure A4).

#### 3.4.3 Potential moderators

The meta-analysis also allows us to consider important dimensions of heterogeneity (Table 5). We consider program intensity (i.e., number of days of training), scale (i.e., number of schools targeted), years between program and outcome measures, and GDP per capita. The first three moderators are motivated by key areas commonly considered critical for impact, and the latter by the differences in self-reported school responsibility and the focus on LMICs in this special issue. Although other moderators are of interest, data limitations prevent us from expanding this set.

A common prior is that programs that spend more time with the principal will have more impact. However, we fail to find support for this hypothesis: the intensity of the training, as measured by the number of training days, does not correlate with greater gains in student learning (Table 5, column 3). Given the time constraints of school leaders, this suggests that an effective program may not require more days of training for the program to be effective at increasing student learning.

We next explore whether the scale of the intervention correlates with impact on student learning. There is a general concern that interventions in smaller studies are delivered with greater intensity, monitoring, and resources, and that this drives impact that will not be replicated for interventions at larger scale (Crawfurd et al., 2022, List et al., 2021). However, we do not see evidence of this concern from the interventions evaluated. Interventions implemented at larger scales yield effect sizes similar to those implemented with a relatively small number of schools.^15^

We then see whether effects are moderated by the time between the intervention and when student learning is assessed, in line with arguments from de Hoyos et al., 2020, de Hoyos et al., 2021 that management interventions take time to translate into performance gains. We find a positive, relatively large, but not statistically significant coefficient (Table 5, column 5). However, when controlling for the other moderators, the coefficient on years to outcome becomes statistically significant at 10 percent.

Finally, we see no statistically significant correlation with country GDP per capita. We therefore fail to find evidence that returns from efforts to improve school management may be more constrained in low-capacity contexts. We may lack sufficient variation to estimate this correlation, however, because our sample includes only three comparisons from low-income countries (from Madagascar and Gambia). Moreover, the null result may mask heterogeneity within countries. For example, though Blimpo et al. (2015) fail to find average learning gains in Gambia, they find that this was not the case in all communities. In communities with higher levels of baseline human capital, as measured by adult literacy, there were learning gains from the program.

Results are similar when including all potential moderators as covariates (column 7). Given that scale, intensity, time, and income do not appear to moderate effects, it is unclear what features are driving the positive average impact we observe. This suggests that these measures may be too coarse to identify key elements that make a program successful, such as who is targeted, what areas are targeted, what materials are provided, and so on. Moreover, these results are correlational, based on a small number of studies for each moderator, and the moderators are likely confounded with other factors. For example, if training intensity is higher in places where institutional quality is lower, then the lack of a correlation may not reflect absence of a causal effect of intensity, but rather the selection of where such programs are implemented. Understanding the drivers of program effectiveness using more rigorous designs is a potential area for future research.

**Table 5:** Regression of effect size on study characteristics.

	(1)	(2)	(3)	(4)	(5)	(6)	(7)
	All	LMICs	All	All	All	All	All
10 days of training			0.004				−0.007
			(0.009)				(0.010)
Schools (’000s)				0.011			0.015
				(0.022)			(0.019)
Years to outcome					0.026		0.029*
					(0.017)		(0.017)
Log GDP pc						0.006	0.012
						(0.006)	(0.008)
Constant	0.033***	0.030*	0.033***	0.031***	0.035***	0.032***	0.033***
	(0.011)	(0.017)	(0.011)	(0.011)	(0.010)	(0.012)	(0.012)

N (Estimates)	56	36	56	56	56	56	56
N (Studies)	20	15	20	20	20	20	20

Note: * p < 0.1, ** p < 0.05, *** p < 0.01. Standard errors in parentheses. This table shows the result from a random-effects meta-regression across the 56 estimates from our 20 studies. The outcome variable is the estimated effect size of the program on test scores. Control variables are all centred at their mean, so that the constant can be interpreted as the average effect across all studies. We use inverse-variance weights so more precise studies are given more influence, and the Hedges et al. (2010) estimator to account for the dependence from when there are multiple estimates from the same study. Column 2 shows results excluding studies from high-income countries.

### 3.5 Barriers to effectiveness

Given the limitations of a quantitative analysis of heterogeneity across programs, we complement this exercise with a qualitative assessment of the barriers discussed in evaluations. In general, many studies included a discussion on factors that reduced the potential of student learning gains. In this section, we highlight some commonalities in the authors’ explanations on why programs were not more effective. We find three common concerns: low take-up among principals in attending the intervention, low incentives or capacity for the principals to adopt the intervention, and the length of the causal chain between intervening on school management to student learning.^16^

Between the initial intervention targeting a school leader’s management to the final outcome of student learning, several intermediary steps allow for potential student performance gains to be realized. First, the principal must participate in the intervention. Second, the principal must have the capacity and incentives to implement the intervention provided. If either are incomplete, then this will reduce the potential impact of the intervention on student learning. Studies included in our review commonly noted low take-up and low adoption as reasons for limited downstream impacts on student learning. The authors also highlighted that the methods to improve take-up, implementation capacity, and incentives for school leaders to incorporate what they learned were critical to help realize the potential of these interventions.

Most of the comparisons used in this review estimate an intent to treat (ITT) effect of the intervention, i.e., the offer of the intervention. This is an important estimate in determining cost-effectiveness, and the returns to an intervention at scale. But equally important is the effect of the intervention conditional on the principal participating and adopting the program, or average treatment effect on the treated (ATT). The gap between the ATT and the ITT estimate identified by the reviewed study is often the decision of the principal to participate in and adopt the program.

In many of the studies, school leaders simply did not attend the training sessions, suggesting a large gap between ITT and ATT. For example, Kraft and Christian (2022) study the effect of providing trainings on offering teachers feedback, but only 60% of the principals who were offered the trainings attended at least one session. Similarly, in Ganimian and Freel (2020), only 69% of the treated schools attend the intervention. In several studies in Argentina (de Hoyos et al., 2020, de Hoyos et al., 2021), take-up of the capacity building workshops were variable and difficult to sustain. Romero et al. (2022) find that the already low take up of their intervention fell to nearly zero when the program was implemented through a “training of the trainers” method, a common approach to delivering interventions.^17^

Even in cases where school leaders participated and engaged in the intervention, not everyone adopted the recommended practices. Some studies noted that the schools were often not provided the capacity or incentives to follow through. In addition, if implementation of school improvement plans or managerial skills are time consuming or have a high fixed cost, then without the proper incentives or accountability structures, school leaders may decide not to follow through. For example, interventions providing diagnostics or encouraging school improvement plans often did not provide a structure or support in how the school leader should follow up. In one such intervention in India, qualitative interviews with principals and others revealed that completion of the school improvement plan fulfilled an administrative requirement and was viewed as a data collection exercise; once the school improvement plans were submitted to authorities, the program was considered completed (Muralidharan & Singh, 2020). In the US, Kraft and Christian (2022) note that the principals lacked sufficient time to implement training on how to provide instructional feedback to teachers.

A third explanation common to several studies was the length and fragility of the causal chain linking school management training to student performance, as in an O-ring production function (Kremer, 1993). Principal attendance at the trainings and implementation of training lessons is not always sufficient for achieving downstream effects on student learning. For example, Ganimian and Freel (2020) note that small effect sizes on student learning may reflect the long path from the intervention to student learning, and de Hoyos et al., 2020, de Hoyos et al., 2021 hypothesize that management interventions take a long time to translate into performance gains. The structure of school accountability to support principals – including parents, school management committees, inspectors, and ministries of education – may also need to be aligned to promote learning (Kaffenberger and Spivack, 2023, Mbiti, 2016). Without changes filtering down to classrooms, either through personnel changes or engaging existing teachers to improve instruction, changes in management practices alone are unlikely to improve student performance.

## 4 External validity and gaps in the evidence base: How do evaluated interventions compare to programs globally

How comparable are the school management interventions included in our systematic review to the programs currently implemented by practitioners? To answer this question, we conduct a survey designed to capture key features of school leader training programs. This is a simplified version of the “In-Service Teacher Training Instrument” (Popova et al., 2022), designed to capture important features of teacher training programs, and adapted for use with school leaders by Adelman and Lemos (2021). We apply our instrument to three groups of programs; first, programs included in our systematic review; second, potentially innovative programs implemented by NGOs; and third, programs implemented at scale by governments. Our sample of evaluated programs comes from our systematic review in Section 3. Our sample of potentially innovative NGO programs is provided by a convening organization, Global School Leaders, with ties to local school leadership NGOs in LMICs throughout the world. Our sample of at-scale government programs is drawn from a list of World Bank programs identified by Muralidharan and Singh (2020). World Bank programs are typically negotiated with, and implemented in close collaboration with the government. We contacted each of the study authors, NGO leaders, and World Bank Task Team leaders, with a request to complete the survey. In cases where we did not receive responses, we also attempted to complete the survey using publicly available project documentation.

Our final dataset includes 12 (of 20) evaluated programs, 10 (of 23) potentially innovative NGO programs, and 13 (of 34) large-scale World Bank-supported government programs. The relatively high non-response rates are clearly a limitation, with the direction of any resulting bias unclear. We nonetheless think this is a useful starting point to assess gaps between evidence and practice.

We present descriptive statistics from this survey in Table 6, in the form of medians to reduce the influence of outliers. First, the scale—measured as median number of schools—of evaluated programs (213) is less than NGO (550) and government (1420) programs. We see a similar pattern in the total number of school leaders targeted, in which evaluated programs cover fewer leaders than NGO and government programs. Thus, the evidence base generally includes smaller programs than those implemented by practitioners.

In contrast, the intensity and labor inputs (measured in median weeks and hours) of evaluated programs is within the range of these measures for NGO and government programs. NGO programs are considerably more intense than their government counterparts: 52 relative to 2 weeks, and 80 relative to 24 h. Evaluated programs run for an median of 64 h over 4 weeks. The median leader to trainer ratio in evaluated programs (17.6) is also within the range of NGO (17.5) and government programs (10). Though NGOs have more time with leaders as measured by hours and weeks of the program, government programs are more intensive in their use of trainers.

Evaluated programs are cheaper ($100), in median cost per trainee, than both NGO ($400) and government ($1008) programs. Evaluated programs remain the cheapest even when adjusting for the GDP per capita of the country where the program is implemented, though by this metric NGO programs are most expensive. The cost advantage of evaluated programs is surprising, as the larger scale of the NGO and government programs should also reduce their cost per trainee. It is also troubling, because any diminished effects at scale would not be offset by reduced costs.^18^

Finally, to measure training quality, we use a checklist of 25 high quality practices based on the World Management Survey (Bloom et al., 2015). The practices include target-setting, systems for monitoring performance, and staff management. Evaluated programs use a median of 70 percent of these practices, similar to NGO programs (82 percent), but much more than government programs, which use only 24 percent. The evidence base therefore reflects practices closer to NGO programs than to government programs.

**Table 6:** External validity of evaluated programs.

	Evaluated	NGO	Gov	All
Total schools	213	550	1420	450
Total leaders	329	1151.5	686	775
Total weeks	4	52	2	4.5
Total hours	64	80	24	75
Leaders per trainer	17.58	17.5	10	13.25
Cost per trainee (USD)	100	400	1007.5	375
Cost per trainee (% GDPpc)	7.47	27.7	19.36	19.21
Share of high quality practices	70	82	24	56
N	12	10	14	36

Note: Table reports medians per program. Cost per trainee (% GDP pc) reports median of program cost as share of country GDP per capita, using GDP per capita in the country of program implementation. Studies in column 1 include (Beg et al., 2021, Blimpo et al., 2015, de Barros et al., 2019, de Hoyos et al., 2020, Fryer, 2017, Ganimian and Freel, 2020, Jacob et al., 2015, Kraft and Blazar, 2014, Lassibille et al., 2010, Lohmann et al., 2020, Romero et al., 2022, Tavares, 2015).

## 5 Conclusion and directions for future research

The literature using modern methods to measure and improve school management practices is burgeoning, but remains in its infancy. Although our review focuses on low- and middle-income countries, this observation applies equally to high-income countries. Applications of the World Management Survey to the public sector and developing countries are scarcely a decade old. Evaluations of management reforms and training, relying on experimental or quasi-experimental methods to identify causal effects, have also emerged only in recent years. Our meta-analysis provides a useful aggregate, but is based on relatively few studies, all of which are considerably heterogeneous in context and programming. Attempts to draw lessons from the aggregated analysis must therefore apply the caution appropriate to a new and quickly evolving body of evidence.

The meta-analysis reveals 0.033 sd in learning gains. This estimate is statistically significant and robust to several alternative specifications. At first glance, these learning gains may appear small. However, the diffusion of these learning gains throughout a whole school exerts a powerful influence on cost effectiveness. Our meta analysis implies cost effectiveness of 2.1 sd cumulative learning gains per $100 for the median program.^19^ This estimate places in the middle range of cost effectiveness among education interventions reported by Kremer et al. (2013). This estimate is also arguably conservative, as it only considers effects after one year despite some evidence of effect persistence (de Hoyos et al., 2020), and uses the more expensive cost estimates of NGO and government and programs ($400 per trainee) rather than the cheaper evaluated programs ($100; Table 6).

Additionally, the recurrence of low take-up and adoption among evaluated programs suggest that improving these dimensions could result in further increases in student learning. However, the heterogeneity of the programs makes it difficult to identify which factors led to successful interventions to improve school management.

Thus, much work remains to understand how to improve the effectiveness of school management programs targeting school leaders. We highlight that reduced take up and follow up structures appear to inhibit the potential effects on student learning. Thus, a key remaining question is: what factors could increase program take-up and adoption of better management practices by school leaders?

In addition, future research should explore which design elements are most influential for impact and cost-effectiveness. A first, basic step, is to report program costs, which appear in only a few studies included in our review. We were therefore unable to explore the relationship between program cost and impact across studies and relative to other methods to improve school performance. Only a handful of studies explore different design elements, such as (Romero et al., 2022) (direct training v. train the trainers in Mexico) and Beg et al. (2021) (adding training in people management to training on differentiated instruction in Ghana). Evidence on *teacher* training shows that programs linked to career incentives are more effective (Popova et al., 2022). Yet no intervention covered in our systematic review evaluated the role of accountability or incentives for principals to improve management practices or other outcomes, either as a carrot (e.g., increased salary, promotion, or school resources) or stick (e.g., school or principal sanctions). Much exploration of design elements remains.

The literature can also further probe the theory of change from management training to student learning. Although management training programs usually target the school principal in isolation, the actions of other actors – school inspectors, teachers, students, and households – can influence program impact (e.g., Cilliers & Habyarimana, 2021). Discussions of school management programs often implicitly focus on the *production function parameter*, which holds responses from actors other than the principal fixed (Glewwe and Muralidharan, 2016, Todd and Wolpin, 2003). But RCTs measure the *policy parameter*, i.e., the overall program effect inclusive of all such responses. Management practices do not change in a vacuum. Given the relatively lengthy causal chain between management training and student outcomes, research should seek to disentangle elements of the policy parameter. Our meta-analysis suggests that simple answers, such as program scale or training intensity, are insufficient.

Links between management training, management practices, and teacher activity are analyzed in several studies included in our review. Findings have been mixed, although we are unaware of studies finding reductions in teacher effort in response to management training (as would be the case from crowding out if principal and teacher effort are substitutes). However, strategic responses by households or other agents in response to management changes remain largely unexplored. Does management change crowd in or crowd out effort and investments by households? Such responses can enhance or mitigate program effects, in some cases reversing the positive effects which may otherwise occur (e.g., Lucas & Mbiti, 2014).^20^

Finally, most of the focus of the literature reviewed here has been on student test scores. Research increasingly suggests that much of the long-term value of schooling may be in various “non-cognitive” or “character” skills, and not well captured by short-term test scores (Jackson, 2018). School management could plausibly improve school culture, helping teachers to develop students both character and cognitive skills, and improving students safety and well-being. Few of the studies in our review consider outcomes outside of test scores. Of those few, Ganimian and Freel (2020) find no change in student-reported school climate, and three studies find improvements in student attendance (Lassibille et al., 2010, Lohmann et al., 2020, Tavares, 2015). The two studies focused on reducing school violence (Devries et al., 2015, Smarrelli, 2021) both find changes in reported behavior - an outcome at least as important as student learning. There is much more to learn about whether school leader training can improve student well-being beyond short-term test scores.

## CRediT authorship contribution statement

**Gautam Anand:** Investigation, Writing – review & editing. **Aishwarya Atluri:** Investigation, Data curation, Writing – review & editing. **Lee Crawfurd:** Conceptualization, Investigation, Methodology, Formal analysis, Writing – original draft, Writing – review & editing. **Todd Pugatch:** Conceptualization, Investigation, Methodology, Writing – original draft, Writing – review & editing. **Ketki Sheth:** Conceptualization, Investigation, Methodology, Writing – original draft, Writing – review & editing.

## Uncited references

Duflo et al., 2020, Glewwe and Maïga, 2011

## Data Availability

Data will be made available on request.

## Appendix A Supplementary data

MMC S1 Online supplementary material.

## Footnotes

1 For example, Muralidharan and Singh (2020) identify 84 countries with school management improvement programs funded by the World Bank.

2 We use the terms *school leaders, principals*, and *head teachers* interchangeably.

3 Though the focus of this review is the evidence base for evaluations in low- and middle-income countries, we retain the five additional studies from the United States given the small overall number of studies. All results are robust to their exclusion.

4 A slightly adapted version of the instrument was also used by Adelman and Lemos (2021) to review school leader training programmes in Latin America and the Caribbean.

5 Global School Leaders (Leaders, 2020) also highlight different roles of school leaders and the lack of opportunities for school leaders to improve their management skills.

6 Throughout this paper we categorize countries according to the 2022 World Bank country income classification, which classifies countries with GNI per capita (calculated using the World Bank Atlas method) of $1085 or less in 2021 as low-income, between $1086 and $13,205 as middle-income, and of $13,205 or more as high-income.

7 These sources are the PASEC survey in Francophone Africa, SACMEQ survey in Anglophone Africa, TALIS survey across high and middle-income countries, SDI surveys from 8 low and middle-income countries, and IPUMS census data for 42 countries.

8 We exclude policies that do not engage principals directly, such as system-wide education budget reforms, which could still alter school management practices downstream. For instance, we exclude studies of large-scale privatization in Liberia and Pakistan (Crawfurd and Alam, 2022, Romero et al., 2020).

9 We downloaded search results using the “Publish or Perish” software developed by Harzing (2007).

10 We excluded an RCT testing charter school management practices in the US because it replaced school principals entirely rather than focusing on improving a given principal’s school management skill (Fryer, 2014). We also excluded (Lassibille, 2016) because it examines only management practices, not student learning outcomes.

11 We estimate the random effects model using Restricted Maximum Likelihood, but the results are similar using Maximum Likelihood or Empirical Bayes (Figure A5).

12 For studies that did not report effect sizes in standard deviation, we standardize the effect sizes ourselves using information provided in the paper. Specifically, for Lassibille et al. (2010) we divide the reported effect and standard error by the reported standard deviation of the control group (30). For Garcia-Moreno et al. (2019) we divide the reported effect and standard error by the overall standard deviation of the ENLACE test that is used (100). de Barros et al. (2019) report effects and standard errors in raw test points, and also effects in standard deviations. We therefore calculate the underlying standard deviation as 40 points in math and 48.9 in language, and use this to standardize both effects and standard errors. For Lohmann et al. (2020) we assume that the standard deviation of the test used is 100 points as the mean is 500 points and that is a commonly used test format. We further assume that the standard error is equal in size to the estimate, as the paper reports that the estimate is not statistically significant, and the reported confidence intervals are small. For Jacob et al. (2015), effects and standard errors are shown in raw test points and standardized effect sizes are also reported, thus we again are able to calculate the underlying standard deviation, and use this to standardize the standard error. We then average effects across the three grades that are reported separately. Finally, for Tavares (2015) we divide raw effects and standard errors by the reported standard deviations (37 for math and 55.9 for language).

13 We borrow this approach from Romero et al. (2022).

14 This calculation assumes a simple additive model of learning gains, as in cost effectiveness studies (e.g., Kremer et al., 2013), ignoring any distributional effects.

15 Table A2 notes the number of schools included in the evaluation. Note that the number of schools does not always reflect the sample size used in the estimation, as there may be other treatment arms included in the evaluation. Rather, the number of schools provides a proxy of the scale at which the intervention was being implemented.

16 Additionally, in some studies, reduced effect sizes may reflect methodological limitations. For example, Fryer (2017) argues that principal turnover may explain the fade-out of results in the schools being evaluated. Garcia-Moreno et al. (2019) note that the comparison schools were familiar with the practices that were being encouraged in the intervention.

17 This is a method in which experts train key personnel in the schooling system, and those trained individuals are then tasked with training others.

18 An important caveat is that only four evaluated programs reported costs, giving us less confidence in this cost estimate.

19 In a typical school of 250 students (Walter, 2020), cumulative learning gains would be 0.033 × 250 = 8.25 sd. The overall median cost per trainee across at-scale NGO and government programs reviewed in Section 4 is $400. Translating these costs and benefits into total standard deviations of learning per $100, we have 8.25 sd/$400 × 100 = 2.1 sd.

20 For an example of worker resistance impeding changes in management practices in the private sector, see Macchiavello and Morjaria (2022).
