# Supplementary material for: Improving school management in low and middle income countries: A systematic review
Source: Econ Educ Rev. 2023 Dec;97:None. doi: 10.1016/j.econedurev.2023.102464 (PMC10714127; doi:10.1016/j.econedurev.2023.102464)
Supplement: MMC S1 — Online supplementary material. [file mmc1.pdf]

A. Appendix

A.1. Additional Figures and Tables

Figure A1: Systematic review search process

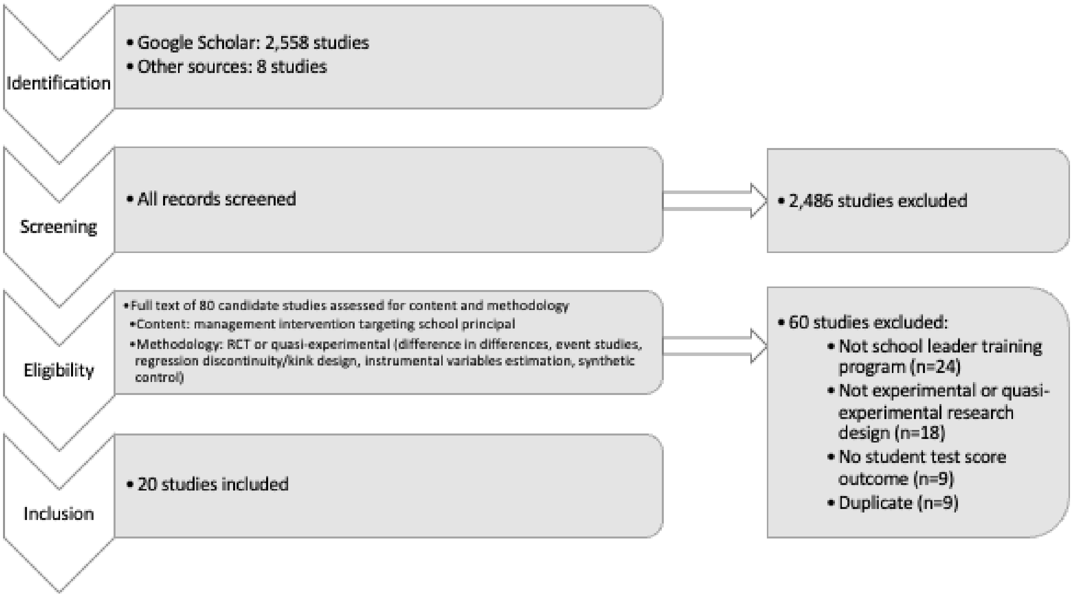

Figure A2: Forest plot of meta-analysis results (all individual estimates)

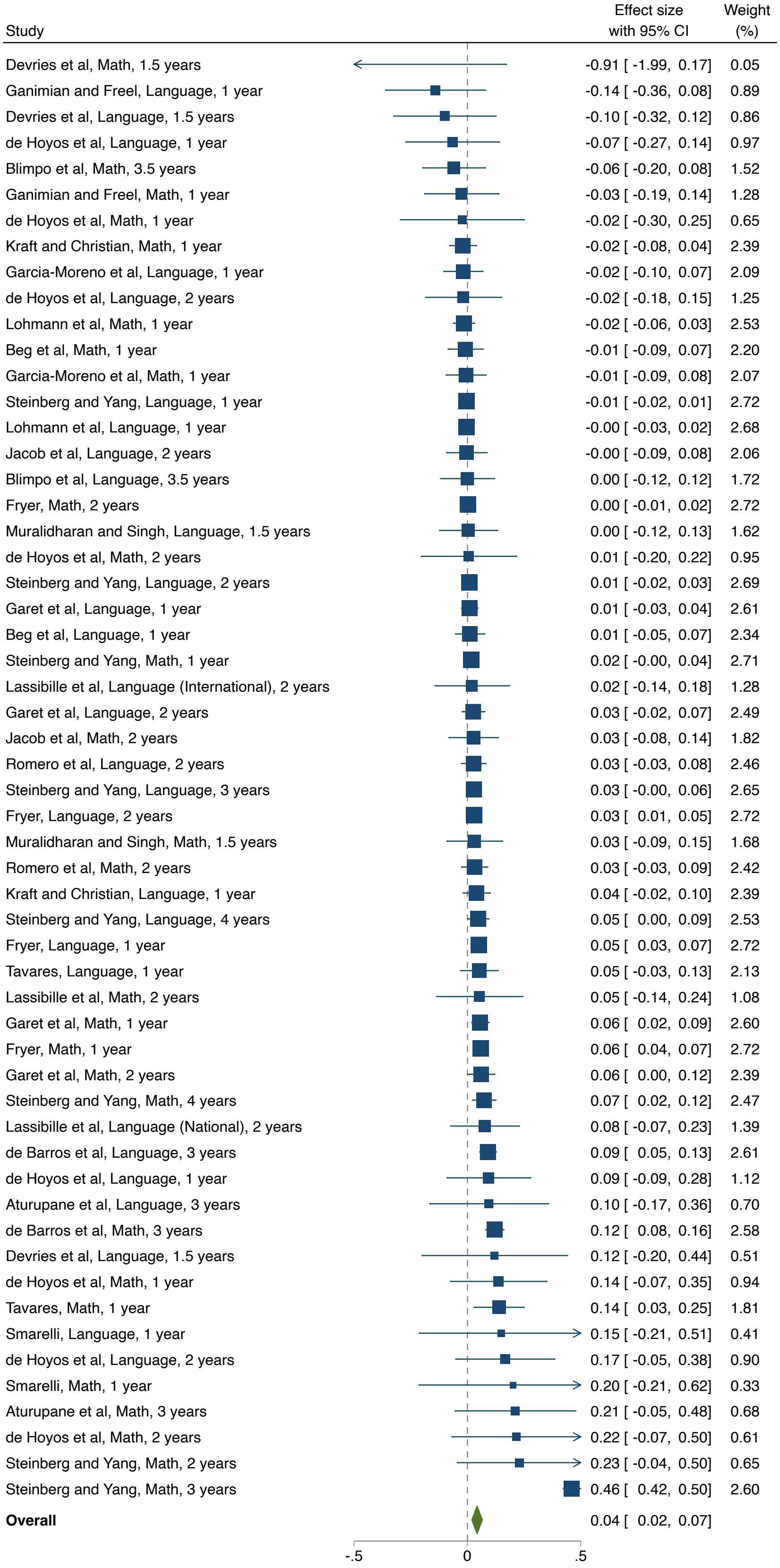

Note: Squares indicate study effect sizes and solid lines indicate 95 percent confidence intervals. Square size is proportional to study weight, which is estimated based on the precision of the estimate. Red diamonds indicate sub-group mean effects, and the green diamond indicates the overall mean effect.

Figure A3: Forest plot of meta-analysis results, by study country income

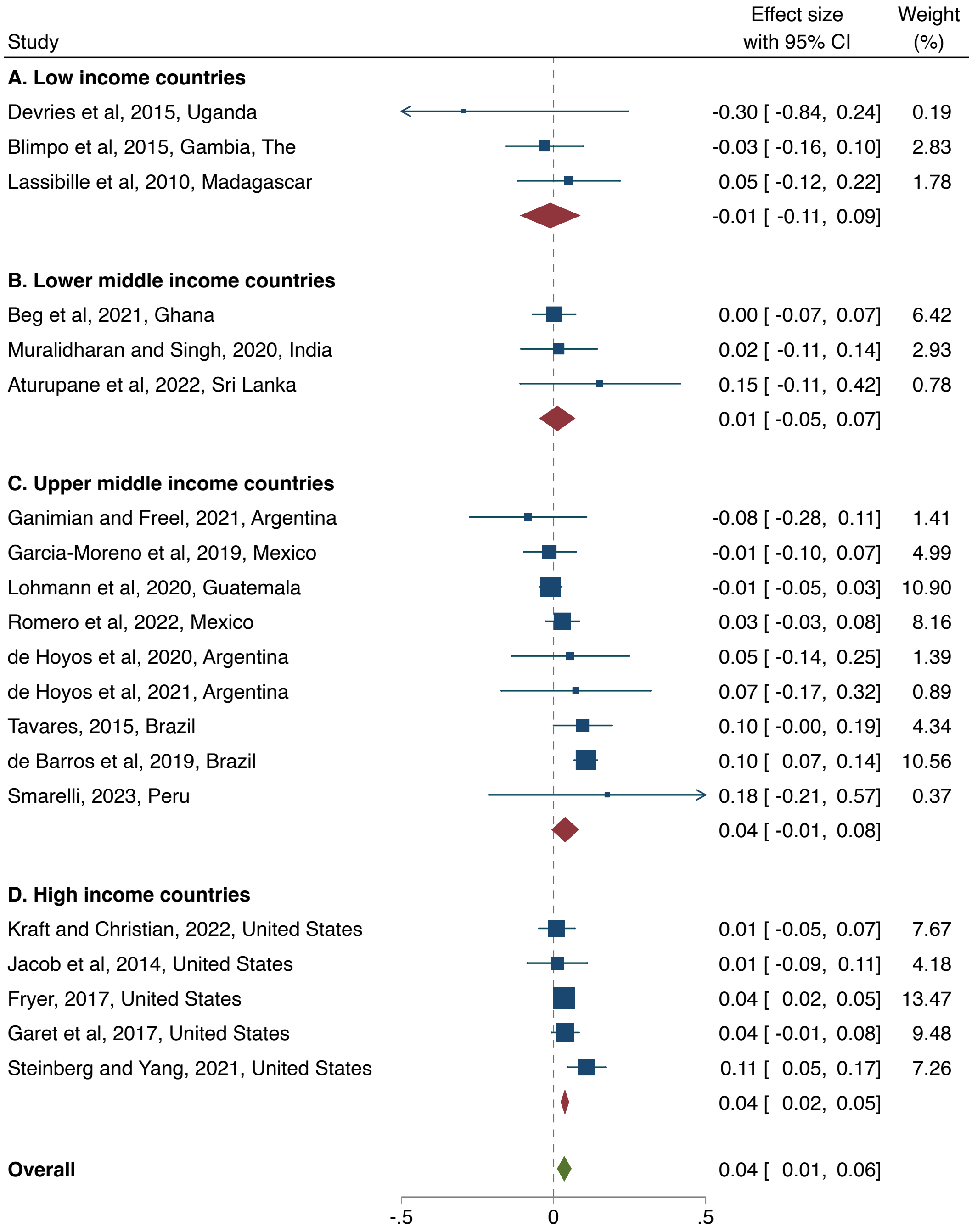

Note: Studies are grouped according to the current World Bank country income classification of their setting. Squares indicate study effect sizes and solid lines indicate 95 percent confidence intervals. Square size is proportional to study weight, which is estimated based on the precision of the estimate. Red diamonds indicate sub-group mean effects, and the green diamond indicates the overall mean effect. Effect sizes and standard errors for each study are both calculated as the mean of individual estimates across different subjects and time periods within each study. This approach is conservative in assuming perfect correlation between estimates within each study, and so providing no increase in precision or weight for studies with multiple estimates (Borenstein et al., 2021). We show all individual estimates in Figure A2.

Figure A4: Meta-analysis robustness to outlier studies

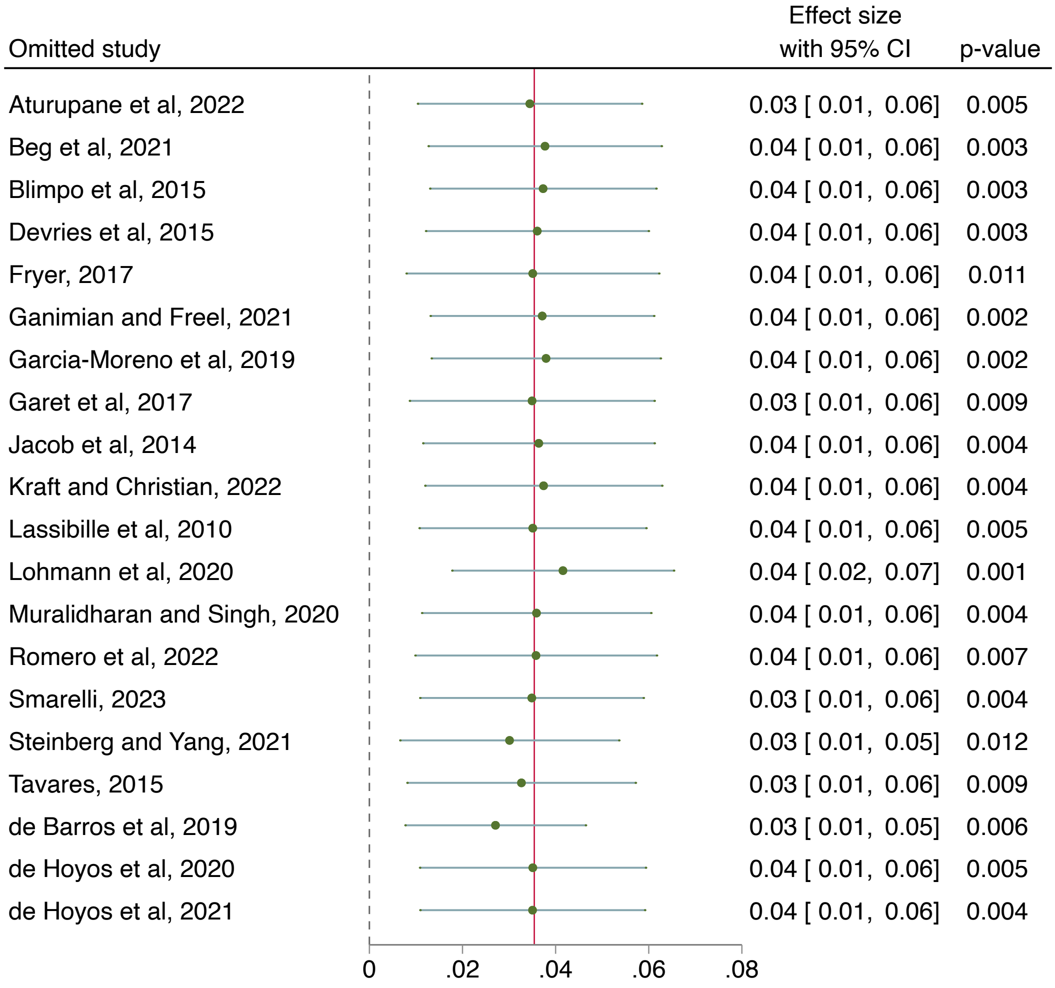

Note: This figure shows the robustness of our main meta-analytic result to leaving each study out one by one. The solid vertical line indicates the overall mean effect size, and the dots and horizontal lines indicate the relevant effect size estimate and confidence intervals for the meta-analysis when sequentially omitting each study. Effect sizes and standard errors for each study are both calculated as the mean of individual estimates across different subjects and time periods within each study. This approach is conservative in assuming perfect correlation between estimates within each study, and so providing no increase in precision or weight for studies with multiple estimates (Borenstein et al., 2021). We show all individual estimates in Figure A2.

Figure A5: Meta-analysis robustness to alternative methods

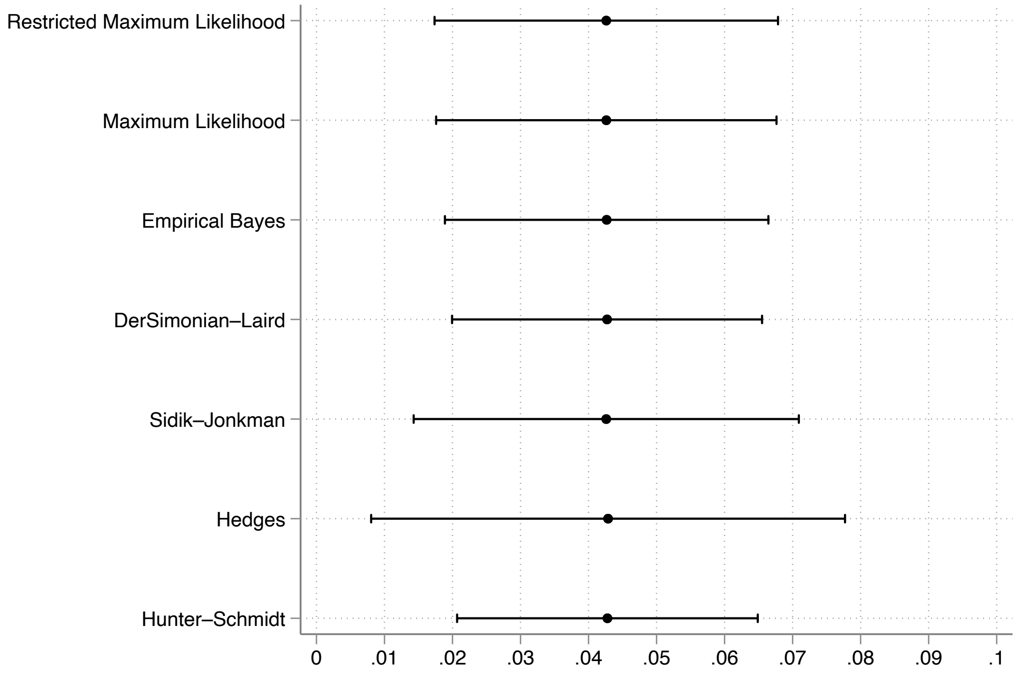

Note: This figure shows the robustness of our main meta-analytic result to alternative estimation methods.

Figure A6: Effect Size Heterogeneity

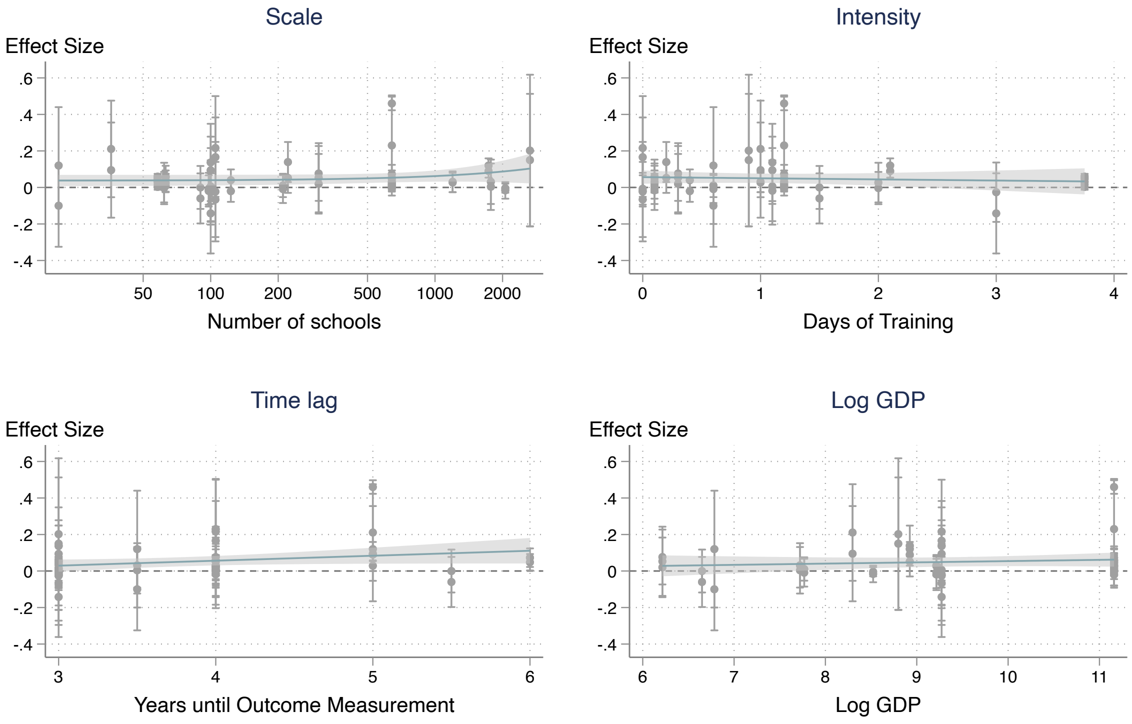

Note: These figures show scatter plots of program effect size plotted against features of the program and context, including the number of schools treated in the program, the number of days of training provided, the number of years between program start and outcome measurement, and the log of country GDP per capita.

Figure A7: Funnel Plot

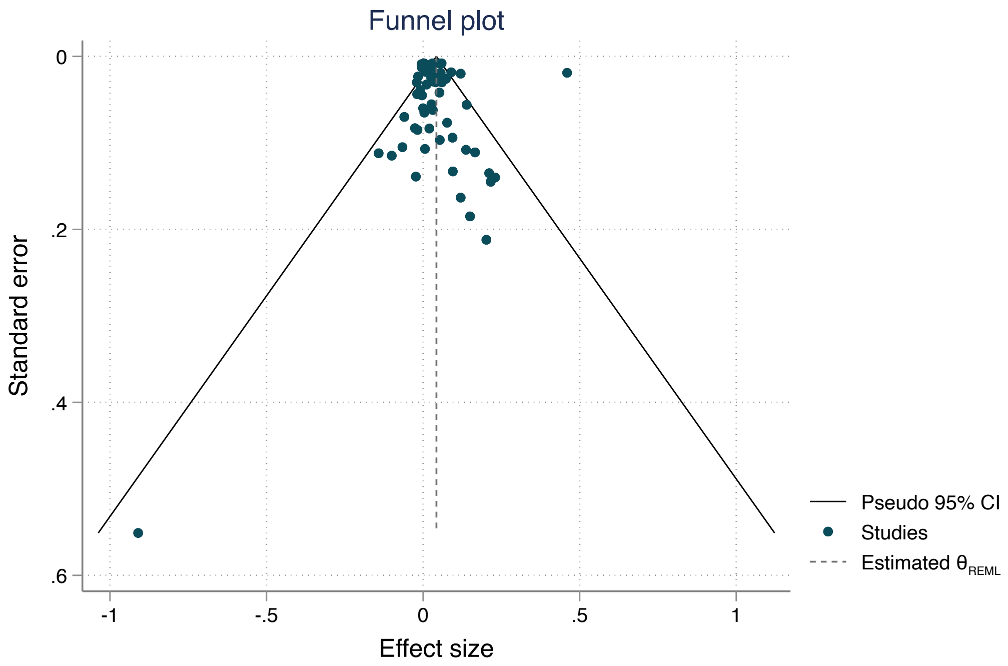

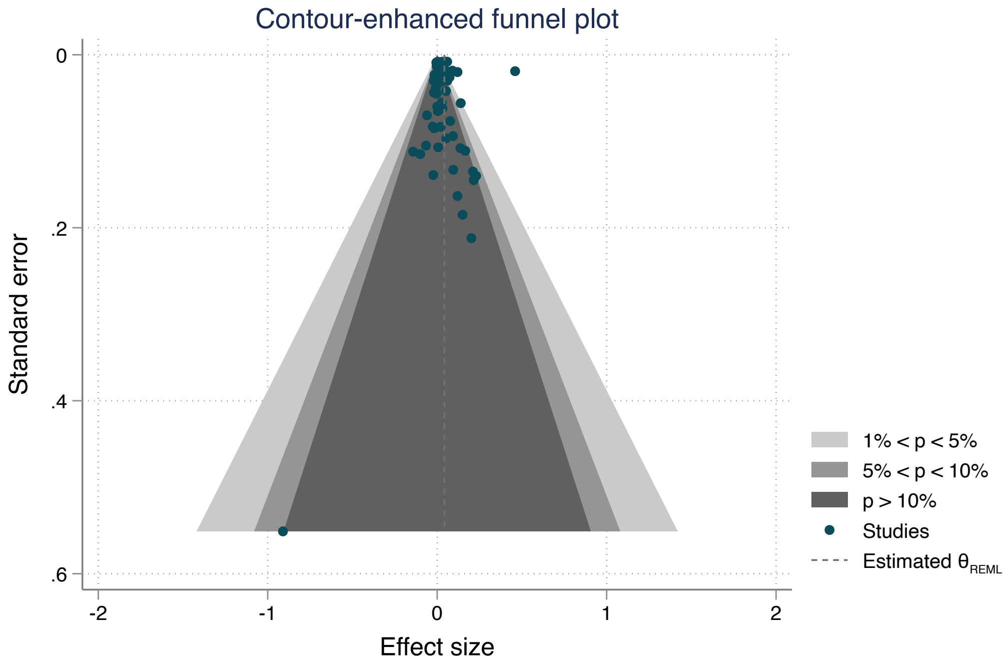

Note: Funnel plots provide graphical tests for publication bias, showing whether more precise studies have systematically different effect sizes to less precise studies. In this case we see no evidence of publication bias.

Table A1: Sensitivity to publication bias

	(1) None	(2) Egger	(3) Non-linear
Effect Standard Error		-0.107	
		(0.432)	
Effect Variance			-1.558
			(2.372)
Constant	0.033***	0.037*	0.037***
	(0.011)	(0.020)	(0.013)
N (Estimates)	56	56	56
N (Studies)	20	20	20

Note: * $p < 0.1$, ** $p < 0.05$, *** $p < 0.01$. Standard errors in parentheses. This table presents two standard tests for publication bias - the Egger regression adjusting for the standard error of each estimate following Egger et al. (1997), or the PEESE approach, adjusting for the variance of each estimate following Stanley and Doucouliagos (2014). In both cases we use the Hedges et al. (2010) estimator to account for the dependence from when there are multiple estimates from the same study.

Table A2: Study Effect Sizes

Study	Subject	Years	Control Group	Effect	SE
Aturupane et al, 2022	Language	3		0.095	0.133
Aturupane et al, 2022	Math	3		0.211	0.135
Beg et al, 2021	Language	1	Teachers trained in	0.011	0.033
Beg et al, 2021	Math	1	differentiated instruction	-0.009	0.039
Blimpo et al, 2015	Language	3.5	Received	0.000	0.060
Blimpo et al, 2015	Math	3.5	mgmt manual	-0.060	0.070
de Barros et al, 2019	Language	3		0.090	0.018
de Barros et al, 2019	Math	3		0.120	0.020
de Hoyos et al, 2020	Language	1	Received information	0.094	0.094
de Hoyos et al, 2020	Math	1	on school performance	0.137	0.108
de Hoyos et al, 2020	Language	2	Received information	-0.018	0.085
de Hoyos et al, 2020	Math	2	on school performance	0.006	0.107
de Hoyos et al, 2021	Language	1	Received diagnostic	-0.066	0.105
de Hoyos et al, 2021	Math	1	information	-0.023	0.139
de Hoyos et al, 2021	Language	2		0.166	0.111
de Hoyos et al, 2021	Math	2		0.216	0.145
Devries et al, 2015	Lang (Int)	1.5		0.120	0.163
Devries et al, 2015	Lang (Local)	1.5		-0.100	0.115
Devries et al, 2015	Math	1.5		-0.910	0.551
Fryer, 2017	Language	1		0.050	0.008
Fryer, 2017	Math	1	Received	0.059	0.008
Fryer, 2017	Language	2	assessments	0.030	0.008
Fryer, 2017	Math	2		0.003	0.008
Ganimian and Freel, 2021	Language	1		-0.142	0.112
Ganimian and Freel, 2021	Math	1		-0.026	0.083
Garcia-Moreno et al, 2019	Language	1		-0.019	0.044
Garcia-Moreno et al, 2019	Math	1		-0.007	0.044
Garet et al 2017	Language	2		0.026	0.025
Garet et al, 2017	Language	1		0.009	0.018
Garet et al, 2017	Math	1		0.056	0.019
Garet et al, 2017	Math	2		0.060	0.030
Jacob et al, 2014	Language	2		-0.003	0.045
Jacob et al, 2014	Math	2		0.027	0.055
Kraft and Christian, 2022	Language	1		0.040	0.030
Kraft and Christian, 2022	Math	1		-0.020	0.030
Lassibille et al, 2010	Lang (Int)	2		0.020	0.083
Lassibille et al, 2010	Lang (Local)	2		0.077	0.077
Lassibille et al, 2010	Math	2		0.053	0.097
Lohmann et al, 2020	Language	1		-0.004	0.013
Lohmann et al, 2020	Math	1		-0.016	0.023
Muralidharan and Singh, 2020	Language	1.5		0.004	0.065
Muralidharan and Singh, 2020	Math	1.5		0.031	0.062
Romero et al, 2022	Language	2	Management training cascade	0.027	0.027
Romero et al, 2022	Math	2	model and same funding	0.031	0.029
Smarelli, 2023	Language	1		0.150	0.185
Smarelli, 2023	Math	1		0.202	0.212
Steinberg and Yang 2021	Language	1		-0.005	0.009
Steinberg and Yang 2021	Math	1		0.017	0.010
Steinberg and Yang 2021	Language	2		0.008	0.012
Steinberg and Yang 2021	Math	2		0.230	0.140
Steinberg and Yang 2021	Language	3		0.029	0.015
Steinberg and Yang 2021	Math	3		0.460	0.019
Steinberg and Yang 2021	Language	4		0.048	0.023
Steinberg and Yang 2021	Math	4		0.073	0.026
Tavares, 2015	Language	1	State mandated	0.052	0.042
Tavares, 2015	Math	1	annual planning	0.139	0.056

Table A3: D-WMS survey instrument: operations management

Topic	Process Implementation	Process Usage	Process Monitoring
	**Questions**		
**1.Standardization of Instructional Processes**	How structured or standardised are the instructional planning processes across the school?	What tools and resources are provided to teachers to ensure consistent level of quality in delivery across classrooms? What are the expectations for the use of these resources and techniques?	How does the school leader monitor and ensure consistency in quality across classrooms?
**2.Personalization of Instruction and Learning**	How much does the school attempt to identify individual student needs? How are these needs accommodated for within the classroom?	How do you as a school leader ensure that teachers are effective in personalising instruction in each classroom across the school?	What about students, how does the school ensure they are engaged in their own learning? How are parents incorporated in this process?
**3.Data-driven Planning and Student Transitions**	Is data used to inform planning and strategies?	If so, how is it used – especially in regards to student transitions through grades/ levels?	What drove the move towards more data-driven planning/ tracking?
**4.Adopting Educational Best Practices**	How does the school encourage incorporating new teaching practices into the classroom?	How are these learning or new teaching practices shared across teachers? What about across grades or subjects? How does sharing happen across schools (community, state-wide etc), if at all?	How does the school ensure that teachers are utilising these new practices in the classroom? How often does this happen?
**5.Continuous Improvement**	When problems (e.g. within school/ teaching tactics/ etc.) do occur, how do they typically get exposed and fixed?	Can you talk me through the process for a recent problem that you faced?	Who within the school gets involved in changing or improving process? How do the different staff groups get involved in this? Does the staff ever suggest process improvements?
**6.Performance Tracking**	What kind of main indicators do you use to track school performance? What sources of information are used to inform this tracking?	How frequently are these measured? Who gets to see this performance data?	If I were to walk through your school, how could I tell how it was doing against these main indicators?
**7.Performance Review**	How often do you review (school) performance –formally or informally– with teachers and staff?	Could you walk me through the steps you go through in a process review? Who is involved in these meetings? Who gets to see the results of this review?	What sort of follow-up plan would you leave these meetings with? Is there an individual performance plan?
**8.Performance Dialogue**	How are these review meetings structured?	Do you generally feel that you do have enough data for a fact-based review?	What type of feedback occurs during these meetings?
**9.Consequence Management**	Let's say you've agreed to a follow-up plan at one of your meetings, what would happen if the plan was not enacted?	How long does it typically go between when a problem is identified to when it is solved? Can you give me a recent example?	How do you deal with repeated failures in a specific department or area of process?
**10.Target Balance**	What types of targets are set for the school to improve student outcomes?	Which staff levels are held accountable to achieve these stated goals?	How much are these targets determined by external factors? Can you tell me about goals that are not externally set for the school (e.g. by the government or regulators)?
**11.Target Inter-connection**	How are these goals cascaded down to the different staff groups or to individual staff members?		How are your targets linked to the overall school-system performance and its goals?
**12.Time Horizon of Targets**	What kind of time scale are you looking at with your targets? Which goals receive the most emphasis?	Are the long-term and short-term goals set independently?	Could you meet all your short-run goals but miss your long-run goals?
**13.Target Stretch**	How tough are your targets? How pushed are you by the targets?	On average, how often would you say that you and your school meet its targets? How are your targets benchmarked?	Do you feel that on targets all departments/ areas receive the same degree of difficulty? Do some departments/ areas get easier targets?
**14.Clarity and Comparability of Targets**	If I asked one of your staff members directly about individual targets, what would they tell me?	Does anyone complain that the targets are too complex? Could every staff member employed by the school tell me what they are responsible for and how it will be assessed?	How do people know about their own performance compared to other people's performance?

Source: https://worldmanagementsurvey.org/data/dwms-public-sector/questionnaires/

Table A4: D-WMS survey instrument: people management

Topic	Process Implementation	Process Usage	Process Monitoring
	**Questions**		
**1.Rewarding High Performers**	How does your evaluation system work? What proportion of your employees' pay is related to the results of this review?	Are there any non-financial or financial bonuses/ rewards for the best performers across all staff groups? How does the bonus system work (for staff and teachers)?	How does your reward system compare to that of other schools?
**2.Removing Poor Performers**	If you had a teacher who was struggling or who could not do his/ her job, what would you do? Can you give me a recent example?	How long is under-performance tolerated? How difficult is it to terminate a teacher?	Do you find staff members/ teachers who lead a sort of charmed life? Do some individuals always just manage to avoid being fired?
**3.Promoting High Performers**	Can you tell me about your career progression/ promotion system? How do you identify and develop your star performers?	What types of professional development opportunities are provided? How are these opportunities personalised to meet individual teacher needs?	How do you make decisions about promotion/ progression and additional opportunities within the school, such as performance, tenure, other? Are better performers likely to be promoted faster, or are promotions given on the basis of tenure/ seniority?
**4.Managing Talent**	How do school leaders show that attracting talented individuals and developing their skills is a top priority? How do you ensure you have enough teachers of the right type in the school?	Where do you seek out and source teachers?	What hiring criteria do you use?
**5.Retaining Talent**	If you had a top performing teacher who wanted to leave, what would the school do?	Could you give me an example of a star performer being persuaded to stay after wanting to leave? Could you give me an example of a star performer who left the school without anyone trying to keep him?	
**6.Attracting Talent / Creating a Distinctive Employee Value Proposition**	What makes it distinctive to teach at your school, as opposed to other similar schools?	If you were to ask the last three candidates would they agree? Why?	How do you monitor how effectively you communicate your value proposition and the following recruitment process?
**7.Leadership Vision**	What is the school's vision for the next five years? Do teachers/ staff know and understand the vision?	Who does your school consider to be your key stakeholders? How is this vision communicated to the overall school community?	Who is involved in setting this vision/ strategy? When there is disagreement, how does the school leader build alignment?
**8.Clearly Defined Accountability for School Leaders**	Who is accountable for delivering on school targets?	How are individual school leaders held responsible for the delivery of targets? Does this apply to equity and cost targets as well as quality targets?	What authority do you have to impact factors that would allow them to meet those targets (e.g. budgetary authority, hiring & firing)? Is this sufficient?
**9.Clearly Defined Leadership and Teacher Roles**	How are the roles and responsibilities of the school leader defined? How are they linked to student outcomes/ Performance? How are leadership responsibilities distributed across individuals and teams within the school?	How are the roles and responsibilities of the teachers defined? How clearly are required teaching competences defined and communicated?	How are these linked to student outcomes/ performance?

Source: https://worldmanagementsurvey.org/data/dwms-public-sector/questionnaires/

A.2. Short summaries and additional details of papers

Lassibille et al. (2010) evaluate an RCT in Madagascar, in which randomly assigned districts were provided a bundle of services to streamline operations, including operational tools and guidebooks and training on their use. Two additional treatment arms, not included in the meta-analysis, directed the intervention at administrators higher than at the school level (i.e., did not include the school leader). These additional treatment arms failed at changing management practices at the school level. The same RCT is also evaluated by Glewwe and Maïga (2011); Lassibille (2016).

Lohmann et al. (2020) evaluate a large-scale (4,124 schools) RCT that distilled the 300-hour program of Fryer (2017) into a single training session focused on "rules of thumb" guidance in Gautamala. Treated schools also received a poster and checklists based on these rules of thumb, and an additional session with Ministry of Education officials promoting these tools. This light-touch approach makes the intervention unique within our review. The program improved management and teaching practices, demonstrating the malleability of school management practices in response to a modest and low-cost intervention.

de Barros et al. (2019) leverage the randomized roll-out of a school governance program targeting high schools that bundled school management training, peer support among school principals, and external monitoring in Brazil.

Blimpo et al. (2015) evaluate a school-based management program bundling training for principals, teachers, and community members with a grant in the Gambia. A second treatment group received the grant only. The study accounts for a large proportion of schools in the country. The bundled intervention increased student and teacher attendance.

Garcia-Moreno et al. (2019) uses a randomized roll-out to evaluate a school-based management program in Mexico that had a similar structure as the Gambian program evaluated by Blimpo et al. (2015). The program encouraged school principals, teachers, and parents to design "School Strategic Transformation Plans" and provided grants and technical assistance to implement the plans. The program was national in scale.

Aturupane et al. (2022) evaluate a school-based management program in Sri Lanka that added teacher training alongside management capacity building for principals.

Muralidharan and Singh (2020) evaluate a school governance reform implemented at scale in the state of Madhya Pradesh, India. Similar to the smaller-scale diagnostic feedback programs evaluated in Argentina (de Hoyos et al., 2020, 2021), this program consisted of detailed school rating scorecards based on an initial audit; development of individual school improvement plans in response to the scorecards, with involvement from principals, teachers, and school management committees; and regular follow-up by government supervisors.

Jacob et al. (2015) evaluate a principal training program using the McREL Balanced Leadership Framework in rural northern Michigan, United States. The program intended "to deliver four different types of knowledge deemed important for improving practices: declarative (knowing what to do), procedural (knowing how to do it), experiential (knowing why it is important), and contextual (knowing when to do it)" (Jacob et al., 2015, p. 3). Treated principals reported using better management practices and feeling more efficacious, though teachers reported no changes in the instructional climate of the schools.

Fryer (2017) evaluate an intensive management training (300 hours over two years) to principals in Houston, Texas. Training focused on instructional planning, data-driven instruction, and observation and coaching. This training was designed in part based on the World Management Survey (Bloom et al., 2015).

Kraft and Christian (2022) evaluate principals trained on providing effective instructional feedback to teachers in Boston. The training encouraged principals to adopt coaching language and provide teachers with specific and actionable feedback.

Ganimian and Freel (2020) evaluate the Program on Leadership and Innovation in Education (PLIE) in Argentina. PLIE was designed by the Varkey Foundation, a UK-based NGO, and adapted for the Argentine context with government input. The program included six weeks of leadership workshops for principals, including training and development of a "school innovation project" for subsequent implementation, and follow-up visits by NGO staff.

de Hoyos et al. (2020, 2021) implement two RCTs test the impacts of providing diagnostic feedback on student skills and building principal capacity to use the feedback to improve performance in Argentina. Both RCTs included two treatment arms, 1) a diagnostic feedback group, including student performance reports and online "dashboards;" and 2) a diagnostic

feedback plus capacity-building group, which received workshops on using the feedback and other practices to improve school performance.

Tavares (2015) evaluates a large-scale program in São Paulo, Brazil that combined elements studied in the Argentine programs (Ganimian and Freel, 2020; de Hoyos et al., 2020, 2021). The intervention combined management training, diagnostics and targets for school performance, and development of school improvement plans.

Romero et al. (2022) compare a management training delivered by professional trainers, versus when the same training is delivered through a "train the trainers" group (i.e., where the professional trainers trained school supervisors to deliver the program). The training program was a successor to that studied by (**?**).

Beg et al. (2021) studies an intervention that offers training for head teachers on people management, differentiated instruction for both teachers and head teachers, and a checklist in basic management practices for head teachers in Ghana. They compare this intervention with one that excludes the training on people management and a pure control (i.e., business as usual). Civil servants from the Ghanaian Ministry of Education implemented the interventions. Compared to the control group, both interventions led to changes in management practices and more engagement among teachers. The intervention that included training on people management further increased measures of people management, as intended. Both interventions successfully raised student test scores and were statistically indistinguishable from each other, suggesting no value added from the people management training. The authors contrast the findings to a separate study in Ghana that provided a similar intervention that trained only teachers and failed to improve learning outcomes (Duflo et al., 2020). One potential interpretation is that this highlights the key role played by head teachers in translating capacity building interventions into learning gains.

Two studies evaluate programs aimed to reduce school violence. Devries et al. (2015) evaluate a behavioral intervention targeting principals and teachers, intended to reduce physical violence against primary school children by school staff. A total of 42 Ugandan primary schools were randomly assigned to receive the intervention or serve as a control group. The study found the prevalence of physical violence, as reported by students, in the intervention schools was significantly lower compared to the control schools. Smarrelli (2021) analyzes the impacts

of a large-scale intervention in Peru aimed at improving school heads' skills to manage school violence. Using a fuzzy regression discontinuity design, the study finds that the intervention led to an increase in reporting violence by eligible schools, but this rise was primarily due to changes in reporting behavior rather than a higher incidence of violence.

Garet et al. (2017) evaluated a program in eight US school districts which introduced teacher and principal performance measures and provided feedback based on these measures. The study found that the performance measures were generally implemented as planned and provided information to identify educators in need of support. The intervention resulted in more frequent feedback for teachers and principals in treatment schools, and it had positive impacts on classroom practice, principal leadership, and student achievement.

Steinberg and Yang (2022) examine the impact of the Pennsylvania (US) Inspired Leadership program, an in-service training program for school principals, on teacher and student outcomes. The study finds that PIL led to increased student math achievement by improving teacher effectiveness, particularly in economically disadvantaged and urban schools.
